# Modulated Optoelectronic Properties of MOF/CNF Bionanocomposite Films for Bacterial Growth Control under Visible Light

**DOI:** 10.1021/acsami.5c04982

**Published:** 2025-05-10

**Authors:** Joab D. Guerrero, Raquel Martín-Sampedro, Ramón Cuadrado, Iván Llano, Eva M. García-Frutos, Pilar Aranda, David Ibarra, María E. Eugenio, Luis Vázquez, Javier Pérez-Carvajal, Margarita Darder

**Affiliations:** † Instituto de Ciencia de Materiales de Madrid (ICMM, CSIC), C/Sor Juana Inés de la Cruz 3, Madrid 28049, Spain; ‡ 54402 Instituto de Ciencias Forestales (ICIFOR), INIA-CSIC, Ctra. de La Coruña, km 7,5, Madrid 28040, Spain

**Keywords:** metal organic frameworks, cellulose nanofibers, grafting, photocatalytic activity, antibacterial activity

## Abstract

The present work reports an experimental and theoretical investigation of tuning the optoelectronic properties of MIL-125-NH_2_ nanoparticles by grafting aromatic molecules. The postsynthetic modification of the MOF MIL-125-NH_2_ with 3,4-dihydroxybenzaldehyde (DBA) resulted in a 23% reduction in the bandgap energy, from 2.71 to 2.08 eV, while increasing the absorbance throughout the visible region of the spectrum, which could be attributed to stabilization due to enol-imine/keto-enamine tautomerism, as supported by DFT theoretical calculations. Pristine and grafted MOFs were assembled into cellulose nanofibers (CNF) for the preparation of functional CNF-based bionanocomposites. The mechanical properties of the films improved, with Young’s modulus increasing from 1.3 GPa in CNF to 7.5 GPa in the film with 20% MOF loading. The potential of the developed materials for photocatalytic antimicrobial therapy was evaluated against *Staphylococcus aureus* (S. aureus). In vitro tests showed that both bionanocomposite films with pristine and DBA-modified MOF remarkably reduced bacterial growth due to the photocatalytic action of the MOF under visible light. The inhibition values were around 58% and 72%, respectively, while minimal inhibition was observed under dark conditions. These initial results support the potential use of the developed bionanocomposite films as wound dressings.

## Graphical abstract

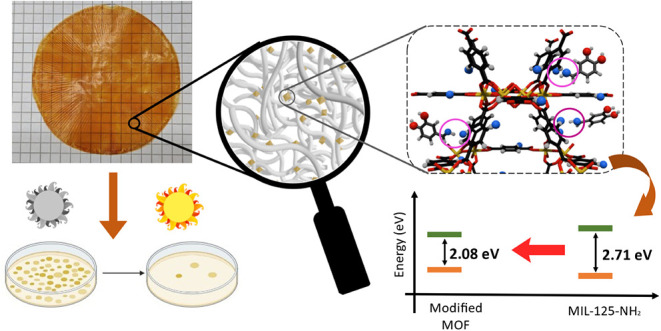

## Introduction

Bacterial drug resistance has become a major threat to modern society, emerging soon after the commercial distribution of the first antibacterial drug in the 1930s.^1,2^ Nonetheless, during what is considered the golden age of antibiotics, it was not regarded as a threat due to the parallel development of new variants of old compounds or the isolation of antimicrobials from natural resources. Over time, the misuse of organic antimicrobial drugs, like penicillin,^3^ has led to resistant strains, significantly reducing the available treatments at present.^2,4^ This fact has stimulated the design and synthesis of new biocidal molecules, such as recently synthesized imidazole analogues and other heterocyclic compounds.^5–7^ Moreover, in the quest for effective treatments to address the problem of antimicrobial resistance, several nanomaterials, such as quantum dots, carbon quantum dots, metallic nanoparticles, metal–organic frameworks (MOFs), covalent organic frameworks (COFs), or MXene nanosheets, among others, hold the potential to revolutionize antimicrobial therapies by overcoming the limitations of current therapies.^8–11^ In particular, nanomaterials exhibiting stimuli–response activity represent a refinement on the new therapies by enhancing efficacy and pursuing the minimization of secondary effects.^10,12^ Photoresponsive materials are a promising solution to address the problem of bacterial resistance through photocatalytic antimicrobial therapy. In this process, photocatalytic materials are excited by light irradiation producing reactive oxygen species (ROS) capable of eradicating a wide variety of pathogens.^13^ For instance, polymeric nanoparticles conjugating photoactive species have proven suitable for visible light-induced disinfection based on the generation of ROS.^14^ Similarly, silver nanoparticles show intrinsic antibacterial response due to the generation of ROS that damage the extracellular membrane, inhibiting the formation of biofilms.^10,15,16^

Among these emerging materials, metal–organic frameworks (MOFs) are a class of advanced porous and crystalline materials formed by the combination of metal atoms or metal clusters and organic molecules.^17,18^ Interestingly, these materials can be prepared in a range of crystal sizes, from millimeters to nanometers. The vast variety of MOFs and their applications^19^ include those focused on providing solutions in the healthcare field, which involve MOFs composed of metals with biocidal properties such as Ag, Zn, Cu, or Co; MOF particles as hosts of antibiotic molecules; or photoactive MOFs able to generate ROS under light irradiation.^20–22^ For instance, zinc-based ZIF-8 MOF has been applied in the health area profiting from its antimicrobial activity^23^ through a mechanism induced by a pH response.^19^ Unfortunately, this MOF presents limited water stability that restricts its application. Alternatively, copper-based MOFs show a catalytic effect in the generation of nitric oxide from NO donors such as S-nitrosothiols, which can also be exploited for biomedical applications that require antibacterial materials,^24,25^ while Ti-based MOFs often present high stability to water and the ability to generate ROS induced by light, as is the case for MIL-125.^23,26^ The activity of this material has been exploited in environmental remediation for visible-light-driven photocatalytic removal of pollutants.^27^ In many cases, MOF photocatalytic activity is enhanced by combination with other semiconductors.^28^ Thus, numerous studies have proven that the formation of heterojunctions between MOFs and other particles, such as silver,^29,30^ palladium,^31^ or silver/palladium nanoparticles,^32^ silver molybdate and silver vanadate^33^ or silver chromate^34^ nanocrystals, copper or iron-based magnetic nanoparticles,^35^ graphitic carbon nitride,^36^ metal phtalocyanines,^37^ metal sulfides,^38^ or even other MOF nanoparticles,^39,40^ can improve the photocatalytic activity under solar light, which can be exploited for a wide range of applications including antimicrobial therapy.

MOF MIL-125 crystallizes as a face-centered cubic topology, whose unit cell is formed by eight Ti octahedral units linked by corner oxygen atoms, obtaining an octamer with a ring-shaped structure.^41^ It presents photocatalytic activity in the UV range due to an optical bandgap of 3.39 eV. To tune its photocatalytic efficiency, isoreticular materials were proposed, like 2-aminoterephthalic acid (BDC-NH_2_), and, in consequence, the new MOF MIL-125-NH_2_ reduced its bandgap down to 2.6 eV, while shifting the photoactivity to the visible range.^42,43^ Moreover, a postsynthetic covalent modification of MIL-125-NH_2_ through its pendant amino units has been proposed to tune the optoelectronic properties. Thus, the amine moieties have been grafted with suitable molecules such as aromatic heterocycles (pyridine-, thiophene-, and quinolone-carboxaldehydes), which provided a reduction of around 2% in the bandgap energy of the final material.^44,45^ Similarly, other MOFs, such as UiO-66-NH_2_,^46–48^ Cu_3_(NH_2_btc)_2_,^49^ MIL-101­(Al)-NH_2_,^50^ or UMCM-1-NH_2_ and DMOF-1-NH_2_ ^51^ have been also subjected to postsynthetic covalent modifications through the pendant amino units for other purposes, grafting molecules such as alkyl and aromatic anhydrides, acetic acid, isocyanates, or 2-methylaziridine.^46–48,52^ In these cases, the effect of the postsynthetic modification was evaluated in terms of the yield increment in different reactions catalyzed by the evaluated materials^47,48,52^ or the tuning of their gas sorption properties.^51^ A recent work by Soilis et al.^53^ addressed the modification of UiO-68-NH_2_ with several aromatic aldehydes, reporting a change in color toward red and a reduction in the bandgap. Similar molecules were selected in the present study for the postsynthetic modification of MIL-125-NH_2_ to tune its electronic bands in comparison to a heterocyclic compound.

The exploitation of this kind of nanomaterial as an antimicrobial skin dressing requires their incorporation into a film. MOFs have been previously combined with biopolymers to form composites in which the particles can provide a reinforcing effect together with the functionality required for a wide variety of applications.^54–56^ Among biopolymers, cellulose is the most abundant polysaccharide on Earth, and cellulosic materials have also proven to be suitable for combination with MOFs to develop new functional materials.^56^ Recently, Tignol et al.^57^ have proven the advantage of MOF/cellulose composites processed as paper sheets, which exhibit high porosity and thus are effective in removing polar volatile organic compounds. Another recent work reports a new MOF-based cellulose acetate material for the photocatalytic preparation of intermediates for numerous industrial products.^32^ Focusing on antibacterial applications, a large number of biopolymer-MOF materials have been developed based on biomacromolecules such as chitosan, silk fibroin, hyaluronic acid, polylactic acid (PLA), or alginate, and also on carboxymethyl cellulose and other cellulose derivatives.^20,58–60^

The specific properties of cellulose, i.e., renewable, biodegradable, biocompatible, and easily functionalizable, make this biopolymer a key material in the transition to the bioeconomy for the production of biofuels, chemicals, and materials.^61^ Cellulose fibers are composed of elemental fibrils interacting through van der Waals bonds and interconnected by hydrogen bonds, forming microfibers. These are hierarchically arranged with other components, forming the cell walls in wood.^61^ These fibrils can be separated by a high-shear mechanical treatment, typically using a high-pressure homogenizer or a microfluidizer, which fibrillates cellulose fibers into high-aspect-ratio nanofibers, with diameters of 5–50 nm and lengths up to a few micrometers.^62^ To promote fibrillation and reduce energy consumption during cellulose nanofiber (CNF) production, chemical, biological, and mechanical pretreatments have been studied, with chemical TEMPO (2,2,6,6-tetramethylpiperidine-1-oxyl radical)-mediated oxidation being one of the most effective pretreatments for these purposes.^63,64^ The resultant CNF films are characterized by properties such as transparency, low density, high mechanical strength, flexibility, thermal and surface stability, and versatility of chemical modification,^65^ which makes them attractive materials for a wide variety of applications,^66^ including antimicrobial applications. Although CNF itself does not exhibit an antimicrobial response, it can capture bacteria due to its high porosity and can be mixed with antimicrobial molecules and nanomaterials that will improve the response against microbes.^67^

Herein, we report an experimental and theoretical investigation of the optoelectronic properties of optimized MIL-125-NH_2_ nanoparticles modified by grafting aromatic molecules onto their lattice through their amine moieties. This modification, which significantly lowers the bandgap energy, aims to shift the nanoparticles’ absorbance, enabling their application across the entire visible spectrum and enhancing the photocatalytic response under solar light with respect to the pristine MIL-125-NH_2_ and other reported postsynthetic derivatives. Subsequently, these nanoparticles were combined with CNF to form a bionanocomposite that can be readily processed into flexible, transparent, and mechanically robust films. In a preliminary study used as a proof of concept, these functional materials were tested against the Gram-positive bacterium S. aureus to confirm their photocatalytic antibacterial activity under visible light and assess their potential application as wound dressings.

## Experimental Section

### Starting Materials and Reagents

Titanium­(IV) (di-isopropoxide) bis­(acetylacetonate) (Ti-ACAC), 75% in isopropanol, was purchased from Tokyo Chemical Industry Co., Ltd. (TCI). 2-Aminoterephthalic acid (BDC-NH_2_, 99%), benzaldehyde (BA, 99%), 4-hydroxybenzaldehyde (MBA, 99%), and 3,4-dihydroxybenzaldehyde (DBA, 97%) were purchased from Sigma-Aldrich. 5-Bromo-1-dodecyl-1H-pyrrolo­[2,3-*b*]­pyridine-3-carbaldehyde (AZD) was synthesized as previously described by Martin et al.^68^ Commercial cellulose from eucalyptus bleached Kraft pulp was kindly provided by the La Montañanesa pulp mill (Lecta Group, Zaragoza, Spain). *N*,*N*-Dimethylformamide (DMF, 99.9%) and pure methanol (MeOH) were purchased from Labbox Labware S.L. Ultrapure water with a resistivity of 18.2 MΩ cm was obtained from a Purelab Chorus 1 System from Elga. All solvents and reagents were used without further purification. All experiments were carried out under an ambient atmosphere.

### Synthesis of MIL-125-NH_2_

The MIL-125-NH_2_ MOF was synthesized under solvothermal conditions by adding 1 mmol of Ti-ACAC 75% in isopropanol as the Ti metal source and 4.5 mmol of BDC-NH_2_ to 10 mL of a DMF:MeOH (8:2) mixture. The reaction time and temperature were set at 150 °C for 24 h. Then, the solids were washed with DMF three times and recovered by centrifugation at 12,000 rpm for 30 min (LISA-R, AFI). All samples obtained following this route were labeled as MIL-125-NH_2_–B*X*, where *X* symbolizes the number of the batch generated.

### Postsynthetic Modification of MIL-125-NH_2_

MIL-125-NH_2_ MOF nanoparticles were grafted by using four different compounds: AZD, BA, MBA, and DBA (Figure 1a). A quantity of dry MOF (65 mg, 0.0381 mmol) was introduced together with the appropriate amount of modifier in a 20 mL reactor chamber, further loaded with 89.86 mg of AZD, 24.26 mg of BA, 27.91 mg of MBA, or 31.57 mg of DBA to prepare MOF-BA, MOF-MBA, and MOF-DBA, respectively, and 15.79 mg of DBA and 7.89 mg of DBA to prepare MOF-DBA_0.5 and MOF-DBA_0.25, respectively. The solids were mixed in 7 mL of acetonitrile (99%) and kept at 80 °C. The powder obtained after 72 h was collected by centrifugation at 12,000 rpm for 30 min and further redispersed in MeOH.

**Figure 1:**
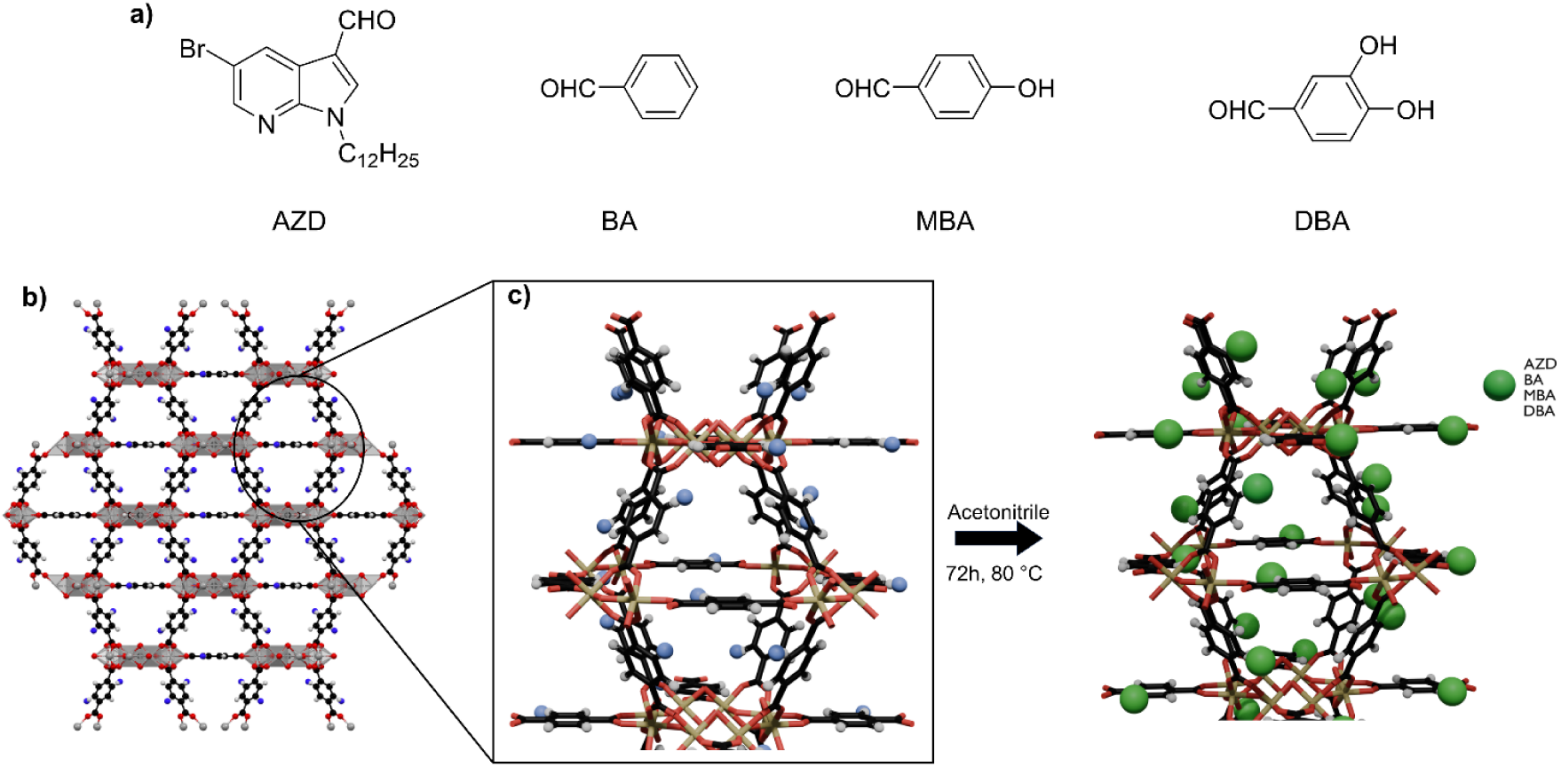
a) Molecules used for chemical grafting to the MOF nanoparticles. b) Schematic representation of MIL-125-NH_2_ Ti-MOF structure. c) Schematic representation of the imine grafting process in the MOF structure.

### Preparation of the CNF and MOF/CNF Films

Prior to the preparation of the bionanocomposite films, cellulose nanofibers (CNF) were obtained from eucalyptus bleached Kraft pulp by TEMPO-mediated oxidation,^63^ as detailed in the Supporting Information. This chemical treatment introduced carboxylate groups at the C6 positions (Table S1) and facilitated mechanical fibrillation through microfluidization (Table S2), leading to an electrostatic stabilization in water and yielding a transparent viscoelastic hydrogel.

Subsequently, MOF/CNF bionanocomposite films were prepared from 0.2% CNF aqueous suspensions with different MOF loadings, ranging from 0% to 32% (Table 1), using a modified filter holder (90 mm diameter, stainless steel) from Millipore, and filtering through a 0.1 μm NUCLPR membrane (Whatman) using compressed air, as schematized in Figure S1. The resulting wet films were placed between two aluminum plates, blotting paper, and a nylon filter mesh with a 10 μm mesh opening and 2% open area (NITEX 03–10/2, Sefar), dried in a vacuum oven at 70 °C for 1 h, and kept under vacuum drying at room temperature for 2 h before use.

**Table 1:** Amounts of CNF and MOF Added in the Preparation of the Bionanocomposite Films by Vacuum Filtration

Samples	CNF (mg)	MOF (mg)	MOF (%)	Grammage (g/m^2^)
CNF	4.160	0	0	19.7
MOF_5%/CNF	4.316	0.210	4.9	21.4
MOF_10%/CNF	3.775	0.374	9.9	19.6
MOF_15%/CNF	3.640	0.514	14.1	19.7
MOF_18%/CNF	3.630	0.677	18.7	20.4
MOF_20%/CNF	3.380	0.817	24.2	19.9
MOF_30%/CNF	3.193	1.051	32.9	20.1

### Characterization Techniques

X-ray powder diffraction (XRD) patterns were collected on a Bruker D8 Advance A25 (DAVINCI) diffractometer with scanning electrons at 40 kV and 40 mA using Cu Kα radiation (λ = 1.5419 Å). The energy dispersive linear detector LYNXEYE XE 500 μm, optimized for high-energy radiation, was used with a maximum opening angle. Fourier Transform Infrared (FTIR) spectra were recorded on a Bruker IFS 66 v/S spectrophotometer, with the samples prepared as KBr pellets, in the 4000 to 400 cm^–1^ region and 2 cm^–1^ resolution. Field-emission scanning electron microscopy (FESEM) images were collected on an FEI Nova NanoSEM 230 scanning electron microscope with a Schottky field-emission gun equipped with a W ion source, using aluminum as a support. Topographic and phase-contrast images were taken by atomic force microscopy operating in the dynamic mode using silicon cantilevers with a nominal force constant of 40 N/m and a radius of curvature close to 8 nm (Bruker). These images were obtained simultaneously with a Nanoscope IIIa system (Veeco). Nitrogen adsorption and desorption measurements were recorded at 77 K by using a 3Flex instrument (Micromeritics). Prior to the adsorption experiments, samples were degassed inside the chamber under a nitrogen flow at 150 °C for 6 h. A selected region of the recorded isotherms was adjusted to the BET model to determine the specific surface area, and pore volume was determined at 0.99 *P*/*P*_0_, both methods assisted by MicroActive software. Titration of the cellulose nanofibers was performed on a Mütek PCD-05 particle charge detector (BTG Instruments) using a 0.001 N solution of poly-DACMAC 99%.

UV–vis spectra were collected using a UV-2401PC spectrophotometer from Shimadzu, employing 1.3 cm diameter flat pills prepared from a solid mixture of MOF nanoparticles and KBr (pelletized at a pressure of 4 tons applied for 4 s). The absorbance and reflectance data were obtained in the wavelength range of 800 to 200 nm and used to calculate the bandgap energy of the materials by applying the Kubelka–Munk approximation for bandgap determination. Tauc plots used in this calculation were generated from the reflectance data measured with the UV–vis spectrophotometer.^69^

### Theoretical Methodology

We carried out geometrical and electronic structure calculations for the bulk metal–organic framework MIL-125-NH_2_ and its grafted derivatives (BA, MBA, and DBA) using density functional theory (DFT) as implemented in the SIESTA code.^70,71^ Core electrons were described using fully separable Kleinman–Bylander and norm-conserving pseudopotentials (PPs) of the Troullier–Martins type.^72,73^ As the exchange-correlation (XC) potential, we employed the generalized gradient approximation (GGA) in the Perdew, Burke, and Ernzerhof (PBE) version.^74^ To account for London dispersion interactions, we applied the D3 correction scheme following Grimme’s formalism.^75^ As a basis set, we used strictly localized numerical atomic orbitals. To achieve a more precise description of the electronic structure and geometries, we performed a self-consistent (SC) optimization of the basis set and PPs cutoff radii for Ti, N, C, O, and H. This was accomplished using an in-house Python-based automated workflow with SIESTA as the core tool and the total SC energy serving as the global optimization variable. The electronic temperature (kT in the Fermi–Dirac distribution) was set to 10 meV. Real-space integrals were computed on a three-dimensional grid with a resolution of 750 Ry, and Gamma-point sampling of the first Brillouin zone provided adequate precision to achieve a SC energy convergence within 1 × 10^–5^ eV. We used the Mulliken partitioning scheme to obtain charge distribution.^76^ Structural optimization was performed by using the conjugate gradient (CG) method at the scalar-relativistic level until interatomic forces were less than 0.05 eV/Å. Atomic positions were allowed to relax within the unit cell, while lattice cell vectors remained fixed at their experimental values.

### Evaluation of the Mechanical Properties of MOF/CNF Films

The tensile tests were performed using an Instron 3345 universal testing machine (Instron Engineering Corp.) with a 100 N loading cell. The samples were obtained from the conformed films and cut into rectangular shapes (Figure S2) of around 1 cm wide with different measured lengths, as indicated in Table S3. The tests were performed at a cross-head speed of 1 mm/min.

### Study of Biocidal Properties of MOF/CNF Films

The antibacterial activity of the conformed bionanocomposite films was studied against S. aureus (CECT 231). Inoculum solutions were prepared by overnight cultivation of one bacterial colony formed in nutrient broth (NB) at 37 °C and 140 rpm. The MOF/CNF films (30 mg) were placed in sterile tubes with 2 mL of sterilized NB culture medium. Controls without films were also carried out. An aliquot (40 μL) of the inoculum was added to each tube, and they were incubated for 2 h at 37 °C and 140 rpm, under irradiation with a LED lamp at 470 nm with a maximum output of 50 W designed and home-built by Dr Alfredo Jacas from the Instituto de Ciencia de Materiales de Madrid (ICMM-CSIC). For comparison, controls with and without MOF/CNF films were also carried out under dark conditions. After the incubation time, serial dilutions of each culture from 10^–1^ to 10^–8^ were performed in sterilized phosphate-buffered saline (PBS). An aliquot (100 μL) of each dilution was uniformly spread on agar medium plates under sterile conditions. After incubation at 37 °C for 24 h, the number of bacterial colonies was counted to determine the inhibition effect, which was expressed as the percentage reduction in the number of colonies, with respect to the control experiment with the CNF film (without MOF).

## Results and Discussion

### Optimized Synthesis and Characterization of MIL-125-NH_2_ MOF and Its Grafting Modification

MIL-125-NH_2_ MOF nanoparticles were synthesized following a modified protocol.^42,77^ In this synthesis, a mixture of BDC-NH_2_ and Ti-ACAC dissolved in dimethylformamide (DMF)/methanol (MeOH) was heated overnight at 150 °C, as indicated in the Experimental Section. The MIL-125-NH_2_ MOF (Figure 1b) was obtained with high reproducibility in terms of crystallinity, porosity, and particle size. The XRD patterns of MOF nanoparticles from three different batches (B1–B3) (Figure S3) match the simulated powder pattern, corroborating that the use of Ti-ACAC results in the expected crystalline phase of MIL-125-NH_2_. The FESEM images show the presence of nanoparticles with a diameter of 180 ± 15 nm with the typical morphology obtained for MIL-125-NH_2_ (Figure S4). These crystalline nanoparticles present permanent porosity, determined by measuring their N_2_ adsorption–desorption isotherms at 77 K (Figure S5), which show apparent surface area values of 1313 ± 68 m^2^/g determined by the Brunauer–Emmett–Teller (BET) method, with a high reproducibility in the BET surface areas obtained for nanoparticles produced in different batches (Table S4).

Postsynthetic modification of the MOF nanoparticles (Figure 1c) was carried out to modulate their optoelectronic properties with the aim of widening their application throughout the entire solar spectrum. The MOF functionalization was based on the grafting protocol described by Wu et al.^44^ using aromatic heterocycles to tune the bandgap energy, which allowed them to achieve a reduction in the bandgap values of around 2%. Similarly, Muelas-Ramos et al.^45^ grafted heterocyclic molecules in MIL-125-NH_2_ that could act as antennas for light absorption, reducing the bandgap by up to 2.6%. Following this approach, we grafted 5-bromo-1-dodecyl-1H-pyrrolo­[2,3-*b*]­pyridine-3-carbaldehyde (AZD) (Figure 1a), as described in the Experimental Section. The selection of this heterocyclic aromatic molecule was based on the prior use of an analogous 7-azaindole derivative to modify TiO_2_ nanoparticles, resulting in a narrowed bandgap.^78^ The FTIR spectrum of MOF-AZD shows a new band at 1701 cm^–1^ that may correspond to the imine bond formation^79^ (Figures 2a and S6). In addition, the band at 1661 cm^–1^ attributed to ν_C=O_ of the aldehyde group in AZD disappears from the MOF-AZD spectrum. XRD powder patterns of the pristine and grafted MOF show differences only in the relative intensity of some planes of the MOF structure, pointing to a high preservation of the structure after chemical modification (Figure 2b). The wider peak centered at 9 degrees can be attributed to a certain change in the symmetry as a result of a partial grafting of the molecules. The corresponding Tauc plot (Figure 2c) shows a 6.6% modification of the bandgap in comparison with that of the pristine MOF, which is slightly higher than the reduction reported by other authors.^44,45^

**Figure 2:**
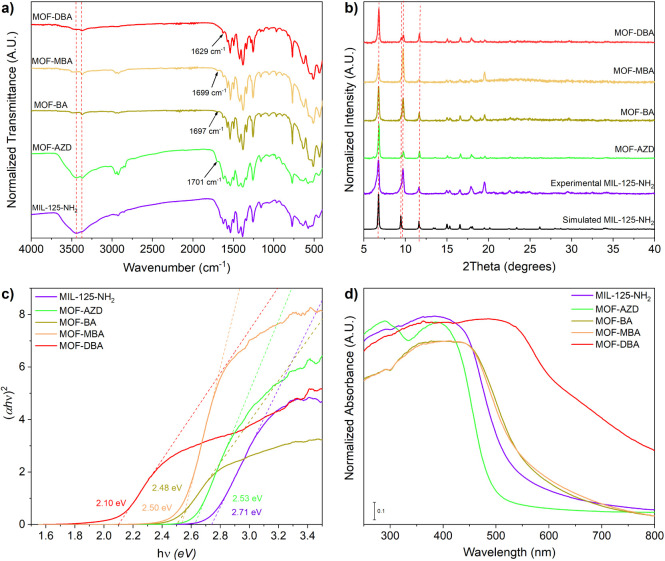
Characterization of MIL-125-NH_2_ samples modified with AZD, BA, MBA, and DBA in comparison with the pristine MOF by means of a) FTIR in the 4000–400 cm^–1^ range, b) XRD, showing also the pattern of the simulated MIL-125-NH_2_, c) Tauc plots for direct bandgap determination, and d) UV–vis absorption.

In order to determine whether aromatic compounds without heteroatoms were able to produce a similar effect, we decided to graft simpler aromatic aldehydes including benzaldehyde (BA), 4-hydroxybenzaldehyde (MBA), and 3,4-dihydroxybenzaldehyde (DBA) (Figure 1a), which present only a benzene ring and an increasing number of hydroxyl groups in order to assess their influence on the optoelectronic properties. The FTIR spectra of these molecules show an intense band at 1700, 1679, and 1647 cm^–1^ for BA, MBA, and DBA, respectively, which is attributed to the C=O stretching vibration of the aldehyde group (Figure S6). As reported, this frequency decreases as the number of hydroxyl groups increases.^79^ These bands are not observed in the spectra of the grafted MOF, corroborating the imine formation. According to previous reports, the stretching vibration of C=N in benzylideneanilines appears at 1633–1624 cm^–1^, but in the spectra of grafted MOF materials, this band could be overlapped by those corresponding to asymmetrical and symmetrical stretching of the carboxylate group interacting with Ti­(IV).^80^ Other changes are observed for those bands at 2819 and 2739 cm^–1^ attributed to the ν_C–H_ of −CHO in the spectrum of BA, together with the reduction of those at around 3400 cm^–1^ due to the stretching vibration of −NH_2_ in the MOF structure, thus confirming the covalent grafting of the aromatic aldehydes as previously observed for AZD. Similarly, the XRD patterns of the resultant grafted MOF (Figure 2b) confirm the preservation of the MOF structure under the tested conditions. Moreover, FESEM images of MOF-DBA (Figure S7) show no remarkable modification in crystal morphology and size compared with the pristine MOF (Figure S4). EDX analysis of both samples shows a slight increase in %*C* from around 41% to 51% due to the grafted molecules. Nitrogen adsorption isotherms of the grafted MOF (Figure S8 and Table S5) show a decrease in the surface area that can be attributed to the reduction of some of the pore cavities due to grafting. BET surface area is reduced from 1267 m^2^/g in the pristine MOF to 941 m^2^/g, 997 m^2^/g, and 831 m^2^/g in MOF-BA, MOF-MBA, and MOF-DBA, respectively. This porosity remains accessible once the molecules are grafted.

After confirming the imine formation and the resilience of the MOF lattice, we determined the optoelectronic modification by applying the Tauc plot determination method. Figure 2c shows a greater modification of the bandgap energy after grafting BA with respect to the AZD molecule. These results show that the modification of the MOF structure with BA reduces by 8.15% the initial bandgap of MIL-125-NH_2_ (Table S6). At this point, we have demonstrated that aromatic rings without heteroatoms can tune the bandgap to the same extension as heterocycles. The MBA molecules presenting one donor moiety produce a similar reduction of the bandgap as observed with BA, while the grafting of DBA with two pending −OH groups gave rise to a substantial reduction of the bandgap by up to 22.5% (Table S6).

Moreover, the grafted MOF also shows a modification of its absorbance properties with respect to nonmodified MIL-125-NH_2_. The grafting of the AZD molecule bearing an electron-withdrawing group reduces the absorbance spectrum of the pristine MOF in the visible range, while the grafting of BA and MBA containing donor groups slightly enlarges the absorption band of this region, and DBA considerably displaces the spectrum to the NIR (Figure 2d). Accordingly, the modification with AZD, BA, and MBA yielded a bright yellow suspension, similar to that of the pristine MOF. In contrast, the modification with DBA turned its color into deep red, pointing to an alteration in the response to light that could modify the photocatalytic activity. To the best of our knowledge, this modification represents the larger modification of bandgap on the MIL-125 MOF and reveals the influence of other factors on the optoelectronic modulation of the energy bandgaps. Considering these results, DBA was selected as the optimal molecule for MOF modification. A detailed study was carried out to evaluate whether and how the reduction of the amount of grafted molecule may affect the MOF properties. For this purpose, various MOF-DBA materials were prepared using different DBA equivalents, and their XRD patterns confirmed the preservation of the crystalline structure of the MOF in all cases (Figure S9). The measured bandgaps are summarized in Table S6, showing that MOF-DBA_0.5 boosts the reduction up to 23.2%, which is slightly higher than the value obtained for the stoichiometric reaction, while MOF-DBA_0.25 already presents an interesting 21.0% reduction of the bandgap energy. These values are higher than those reported by other authors for the grafting of analogous benzaldehyde derivatives to UiO-68-NH_2_,^53^ which also yielded a change in color toward red but a reduction in the bandgap estimated theoretically at around 18% in the best case.

To investigate the mechanism behind the bandgap reduction in the grafted MIL-125-NH_2_ MOF nanoparticles, comprehensive atomistic calculations were conducted on periodic crystals based on density functional theory. The impact of grafted molecules was specifically examined by including BA, MBA, and DBA on the pristine MOF structure. For this purpose, ionic optimizations and electronic structure analyses on MIL-125-NH_2_ were performed along with three modified MOFs: MOF-BA, MOF-MBA, and MOF-DBA. In these simulations, two out of the 12 amine groups of MIL-125-NH_2_ in each unit cell were functionalized, successfully reproducing the band gap reduction observed experimentally. The number of atoms within the simulation cell varies from 256 in the pristine MOF to 286 in the MOF grafted by the DBA molecule. During the optimizations, the unit cell lattice vectors were fixed, allowing only the ions to move freely, and although the high symmetry is maintained, some deformations become apparent upon inspection of the schematic representation of the relaxed structures (Figure 3a) and their simulated XRD (Figure 3b). Interestingly, the structural modification experimentally observed by XRD is revealed after relaxation of the lattice, showing conformational changes on the unit cell that slightly modify the symmetry of the crystalline structure. This modification is clearly assessed experimentally in MOF-DBA (Figure 2b) as a diffraction peak at 9.49 degrees. This effect is not observed in the MOF-MBA diffractogram, which presents an inversion of the intensity of the experimental peaks related to the (101) and (200) planes. This inversion can be attributed to the coordination of the molecule to the titanium cluster through the −OH group, eventually detected in some simulations, which most likely cancels the expected modulation of the bandgap. This could explain why MBA produces a smaller bandgap reduction than BA and no color change in MIL-125-NH_2_, despite having an electron-donating group that is expected to promote electron delocalization, extending conjugation and inducing a bathochromic shift.^53,81^

**Figure 3:**
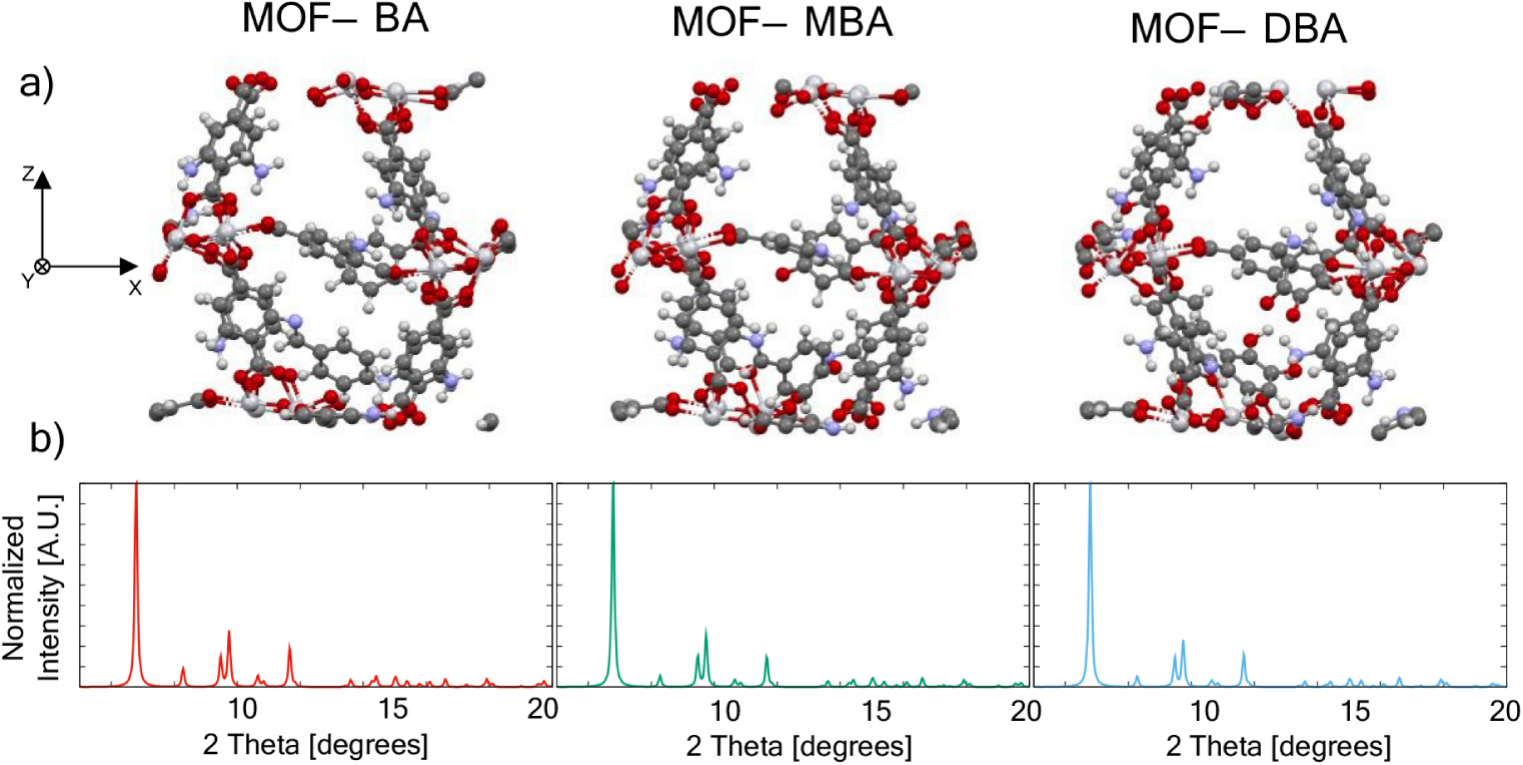
a) Schematic representation of the MOF-BA, MOF-MBA, and MOF-DBA-simulated unit cells after the ionic optimization; and b) simulated XRD of the relaxed crystal structures obtained.

The mechanism behind the band gap reduction can be elucidated through two key factors: first, the local geometric modifications occurring after ionic optimization as a result of functionalization with the BA, MBA, and DBA groups; and second, the analysis of the electronic structure via the density of states (DOS) of these modified structures. In terms of geometry, it is important to note that functionalizing different amine groups as well as varying the number of functionalized groups (fewer or more than two) could result in hundreds of metastable configurations after relaxation. These configurations are influenced by the internal flexibility of certain regions of the unit cells. Addressing all such configurations would require accounting for tens of additional degrees of freedom, which is beyond the scope of this study. Instead, we focused on analyzing a selection of configurations chosen from several trials. These trials involved functionalizing one, two, three, and four of the 12 amine groups in each unit cell with the adsorption sites distributed across different regions of the cage, namely, the upper, middle, and lower sites, depending on where they are adsorbed. Although we optimized and calculated the electronic band structures for all these configurations, only the results for functionalization with two molecules of each grafted molecule (BA, MBA, and DBA) are presented here. The grafted molecules are positioned at the upper and lower inner sites of the pore, as the electronic and geometric behaviors were consistent across all configurations. Interestingly, hydrogen atoms after relaxation show metastable configurations, where the hydrogens initially bond to O and/or C, leading to a keto-enamine conformation.

Figure 4a presents the total density of states (tDOS) for the simulated pristine and modified MOF, showing a band gap reduction from 2.45 eV for MIL-125-NH_2_ to 1.63 eV for MOF-DBA. This trend is in agreement with the experimental results detailed above and also with other theoretical results,^77^ and correlates with the number of −OH groups (two for MBA and four for DBA), the local adsorption sites of these hydrogen atoms, and the charge transfer involving N, C, O, and H. The bandgap reduction also correlates with the different geometric configurations and the emergence of unpopulated states arising from the BA, MBA, and DBA molecules. We anticipate that these states result from the presence of additional C atoms in the MOF structure, the altered chemical environment around the grafted nitrogen atoms, and the charge transferred from these carbon and nitrogen atoms to their surroundings.

**Figure 4:**
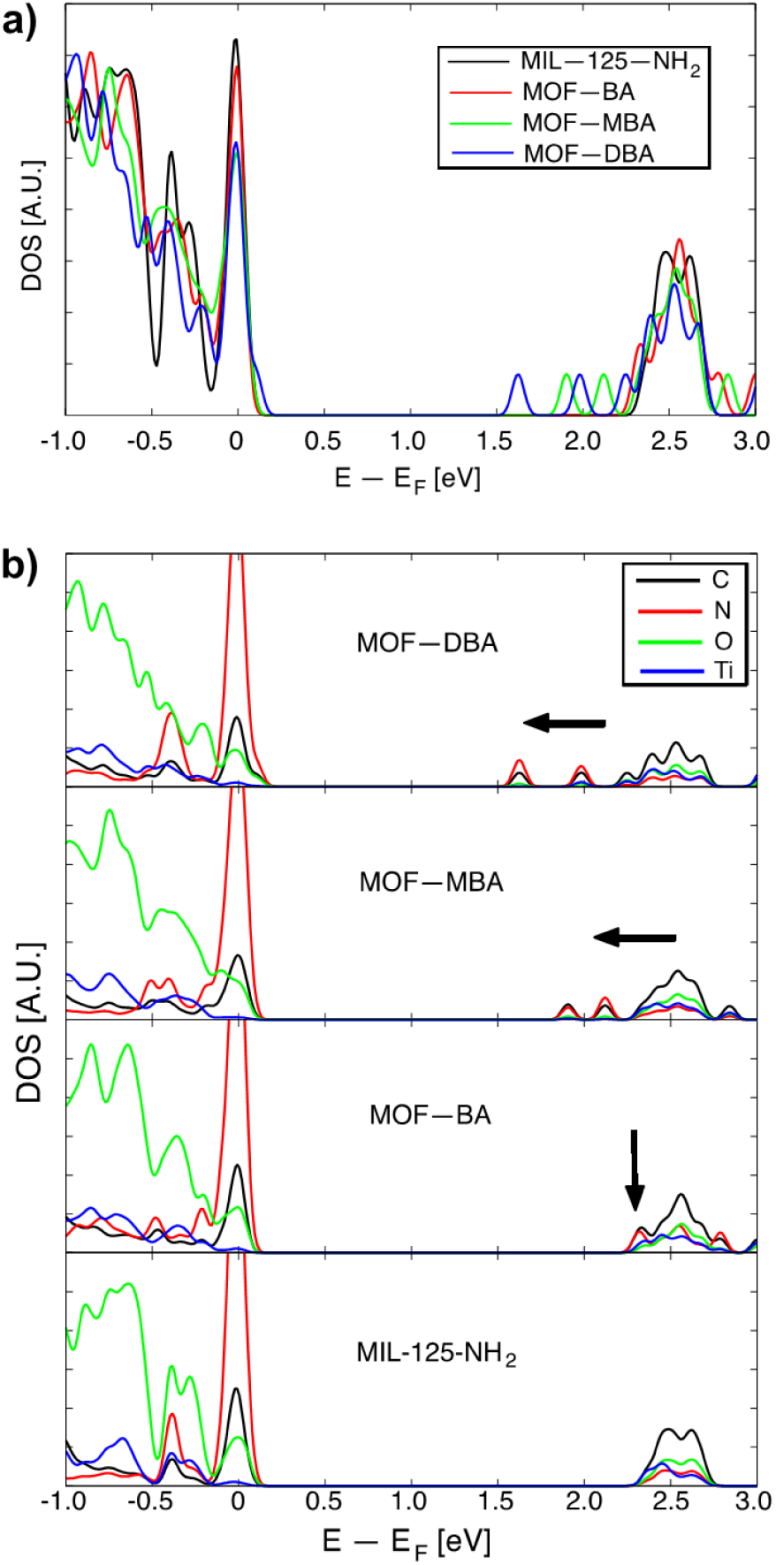
(a) Total density of states (tDOS) of MIL-125-NH2, MOF-BA, MOF-MBA, and MOF-DBA of the optimized crystal structures. (b) Density of states (DOS) projected on C, N, O, and Ti for MIL-125-NH2, MOF-BA, MOF-MBA, and MOF-DBA, shown from bottom to top, respectively. The vertical arrow indicates the first unoccupied states in MOF-BA, while the horizontal arrows serve as visual guides to illustrate the shift of the bands toward the Fermi energy.

Consequently, the electronic structure of the pristine MOF is disrupted, introducing these states within the band gap of the pristine structure and facilitating its reduction. The density of states projected onto the C, N, O, and Ti atoms is represented by the black, red, green, and blue solid lines, respectively, in Figure 4b. The data reveal that the species responsible for the unpopulated states within the band gap are primarily C and N, and, to a lesser extent, O. Additionally, for the MOF-BA configuration, which lacks −OH groups within the moiety, a small hump appears around 2.3 eV, leading to the initial band gap reduction (indicated by the black vertical arrow in the figure). Configurations with one or two −OH groups further shift these bands to lower energy values, resulting in a more pronounced reduction (as shown by the leftward-pointing horizontal black arrows). It is important to note that the C atoms responsible for the appearance of these new bands within the gap are those belonging to the added moieties, along with the N atoms to which they are grafted, while the remaining C and N atoms remain almost electronically unaltered compared to the pristine MOF. A detailed survey of the charge transfer to the environment by N and C atoms shows a −0.3 electron per atom difference, illustrating that both N and C act as donors (Figure S10). However, in the BA, MBA, and DBA configurations, the N atoms that are grafted exhibit reduced donor character compared to their nongrafted counterparts (Figure S10). Notably, in MOF-BA, the nitrogens increase their valence charge by approximately +0.1 electron per atom compared with their net valence charge. All of the C atoms display a similar trend (−0.05 electron per atom), except the C atoms that connect to the amine. It is worth noting that the empty states originated from the C and N atoms almost align their energies, thereby promoting active species within the conduction band (Figure S11). This event strongly points to the relevance of the electronic conjugation of the aromatic system and the high importance associated with the enol-imine/keto-enamine tautomerism typically described in other reticular systems.^82^

### Preparation and Characterization of MOF/CNF Bionanocomposite Films

The use of the obtained materials in wound dressings requires their previous incorporation into macroscopic films that contain active nanoparticles. CNF is an ideal platform for health-related applications as it shows biodegradability, biocompatibility, transparency, and flexibility.^65^ The obtained TEMPO-oxidized CNF presented a charge density of 1.237 ± 0.006 mmol·g^–1^ arising from the carboxylate groups introduced at the C6 positions of cellulose (Table S1), which facilitated subsequent fibrillation through microfluidization. The produced nanofibers were evaluated by atomic force microscopy (AFM), showing lengths close to 1 μm and diameters around 2 nm (Figure 5a). The CNF hydrogel obtained was mechanically mixed with different MOF amounts (Table 1), and then, the mixture was filtered by air pressure to remove water (Figure S1) and finally dried to obtain a homogeneous composite film (Figure 5b). The grammage was set to 20 g/m^2^ to ensure high transparency, allowing the highest percentage of irradiated light to reach the active MOF nanoparticles that will perform the photocatalytic effect. A set of films with different MOF loadings were prepared, showing that for the selected grammage, a maximum of 30% of MOFs was feasible without compromising the bionanocomposite film integrity. XRD patterns of the resulting films show the characteristic planes associated with both components, MOF and CNF (Figures 5c and S12), pointing to some preferential orientation of the MOF particles within the bionanocomposite. In addition, their FTIR spectra (Figure S13) show the characteristic bands related to the pristine components, with an increase in the intensity of those related to the MOF as its loading increases. The absorbance of the films (Figure S14) is due to the embedded MOF, with slight variations corresponding to different nanoparticle loadings. FESEM cross-sectional images (Figure S15) show the high homogeneity throughout the bionanocomposite films.

**Figure 5:**
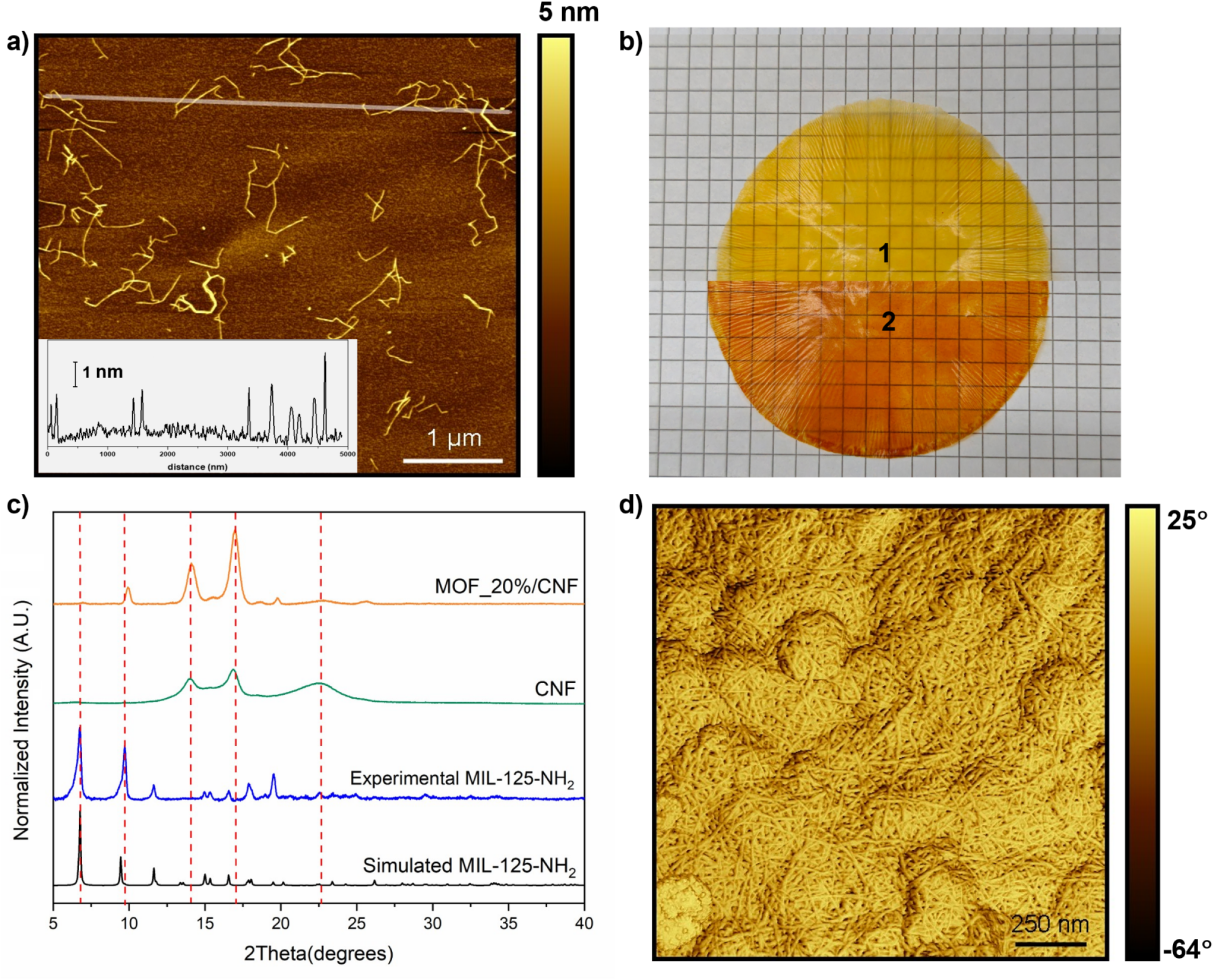
a) AFM image of CNF deposited on mica and profile (inset), corresponding to the straight line. b) Pictures of (1) MOF_20%/CNF and (2) (MOF-DBA_0.5)_20%/CNF bionanocomposite films. c) XRD patterns of simulated MIL-125-NH_2_ (black), experimental MIL-125-NH_2_ (blue), CNF fibers (green), and bionanocomposite MOF_20%/CNF (orange). d) Phase contrast AFM image of MOF_5%/CNF film surface.

In addition, one of the main characteristics sought for the use of these materials as wound dressings is the flexibility and malleability of the films, which will permit their adaptation to irregular wound surfaces, providing also ease of handling and mechanical robustness.^59^ Tensile tests were performed to evaluate the mechanical resistance of the films, showing that the addition of the MOF improves the stiffness and strength of the films (Table 2 and Figure S16). This increase in the Young’s modulus could be attributed to an effective interaction between the nanoparticles and the fibers that produces a reinforcing effect.^83^

**Table 2:** Mechanical Results of the Film Specimens with Different MOF Loadings

Samples	Young’s modulus (GPa)	Elongation at break (%)
CNF	1.3 ± 0.3	6 ± 4
MOF_5%/CNF	2.6 ± 0.6	10 ± 3
MOF_20%/CNF	6.5 ± 0.3	5.1 ± 3
(MOF-DBA_0.5)_20%/CNF	7.5 ± 0.8	3.1 ± 3

The phase-contrast AFM image of this MOF/CNF film (Figure 5d) corroborates that the MOF crystals are embedded in a sort of fiber network, promoting aggregation between the MOF and the fibers during the pressure conformation and improving the mechanical properties of the system.

### Evaluation of the Antibacterial Activity of the MOF/CNF Bionanocomposites

Selected MOF/CNF bionanocomposite films were evaluated against S. aureus, a Gram-positive bacterium responsible for dangerous skin infections,^84^ to assess the potential application of these materials as wound dressings in photocatalytic antimicrobial therapy. Although the antimicrobial effect of MIL-125-NH_2_ powder was proven against S. aureus without light irradiation, possibly due to direct contact and membrane disruption,^85^ other studies reveal its ability to generate ROS due to its photocatalytic activity driven by visible light.^26^ These generated ROS can be exploited for killing bacteria through the oxidative damage of their cellular components. For the antibacterial activity tests, the bionanocomposite films containing 20% of both pristine or modified MOF (MOF_20%/CNF and (MOF-DBA_0.5)_20%/CNF, respectively) were cut into discs 15 mm in diameter. Discs of pristine CNF were also used as a control. Portions of these discs (30 mg) were placed in sterile vials containing the culture medium. After bacterial inoculation, part of the vials was incubated at 37 °C under irradiation with a 470 nm lamp for 2 h. In parallel, the same experiment was carried out under dark conditions for a feasible comparison. After the experiments, the colony forming units (CFU) were determined on agar plates at appropriate dilutions (Figure 6A), showing a clear distinction in bacterial growth reduction between the two sets of experiments, performed in the dark or under light irradiation. The MOF_20%/CNF and (MOF-DBA_0.5)_20%/CNF samples not exposed to irradiation maintained CFU values statistically similar to the control values (Figure 6B). The slight increase compared to the control could be attributed to the presence of CNF, which could be used by bacteria as a nutrient and also as a solid support for their growth, favoring adhesion and biofilm progression.^86,87^ Interestingly, the films loaded with MOF and MOF-DBA showed a similar behavior to the control test, pointing to the absence of toxicity of the MOF and MOF-DBA particles embedded in CNF.^88^ The irradiated CNF film showed CFU values slightly lower than those observed under dark conditions and the control test, although statistically similar to those values. This result could indicate a possible effect of irradiation on bacterial survival. The formation of ROS is expected in the presence of the MOF-containing films, as reported for other MOF-based materials,^23,26^ and a significant decrease in bacterial growth was observed in the presence of the MOF_20%/CNF and (MOF-DBA_0.5)_20%/CNF bionanocomposite films, resulting in inhibition values around 58% and 72%, respectively. Other works have evaluated the inhibition efficiency of MIL-125-NH_2_ against this bacterium. One of them reported inhibition values around 21.36% when using nonmodified MIL-125-NH_2_ and showed that the formation of heterojunctions involving graphene oxide (GO) and Pt nanoparticles^89^ may increase its antibacterial efficiency up to values of 99.94% due to an additional photothermal effect. Similarly, an AgNP/MIL-125-NH_2_ heterojunction was proposed to reach an antibacterial efficiency of 99.98%.^90^

**Figure 6:**
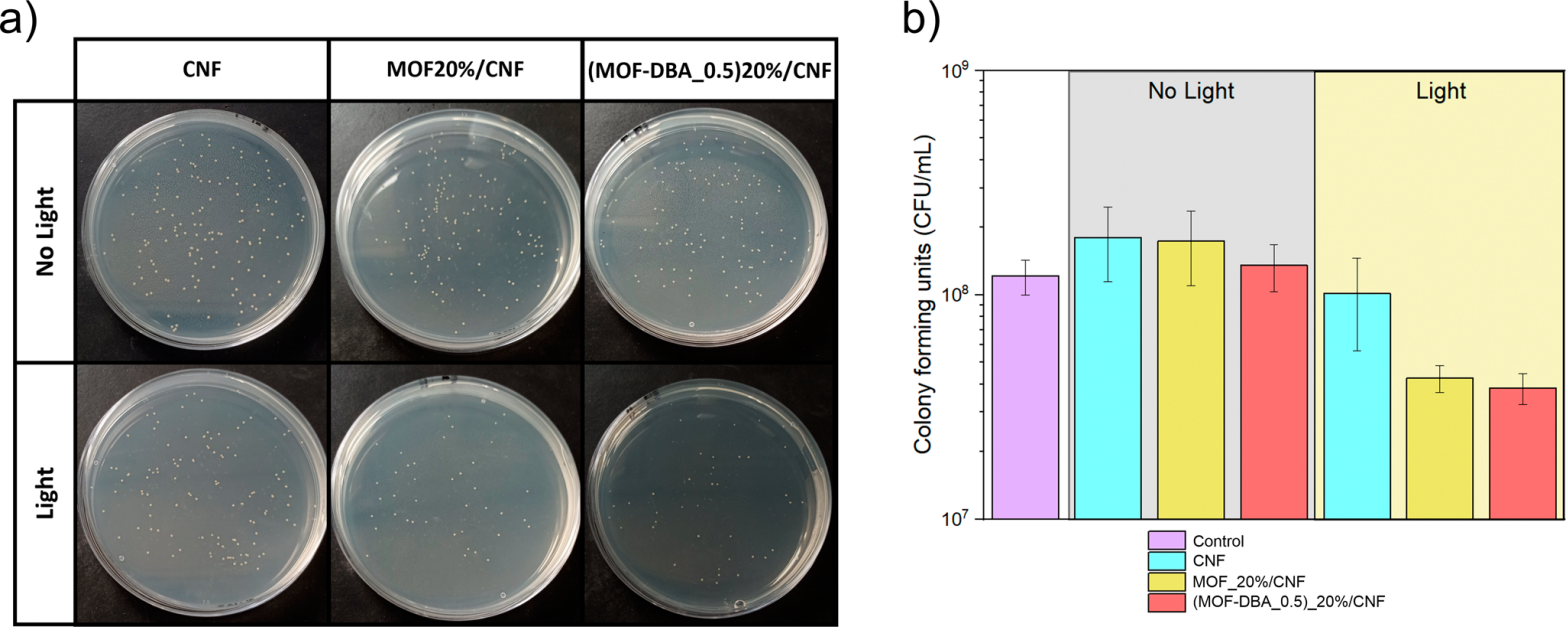
Antibacterial effect against Staphylococcus aureus: (a) colony-forming units at dilution 10^–5^ after treatment with CNF films, MOF_20%/CNF, and (MOF-DBA_0.5)_20%/CNF, under dark conditions (top row) and with light irradiation (bottom row). (b) Average colony forming units for the samples CNF (blue), MOF20%/CNF (yellow), and (MOF-DBA_0.5)­20%/CNF (red) under dark conditions and with light irradiation.

MOF-DBA particles caused a slightly higher inhibition of the bacterial growth compared to the nongrafted MOF, which can be attributed to the reduction of the bandgap and its effect on the generation of additional ROS species.^26^ These results demonstrate that the developed films can be used as a barrier to mitigate the infection by reducing the bacterial cell growth. After the antibacterial tests, the films were recovered and dried, and XRD patterns confirmed that the crystalline structure of the MOF is preserved after exposure to light and the culture medium (Figure S17). This preliminary assay supports the potential use of the developed MOF-based bionanocomposite materials as wound dressings, and their performance could be optimized in further studies.

## Conclusion

We have demonstrated that using titanium­(IV) (di-isopropoxide) bis­(acetylacetonate) (Ti-ACAC) in the synthesis of MIL-125-NH_2_ increases reproducibility in crystallinity, porosity, and particle size, as confirmed by XRD, N_2_ sorption isotherms, and FESEM. We also tuned the electronic properties of MIL-125-NH_2_ by grafting organic molecules to the pristine MOF nanoparticles, achieving a reduction in the band gap values of up to 23% when using 3,4-dihydroxybenzaldehyde (DBA). This effect is supported by DFT simulations of the proposed structures, where the influence of internal flexibility, conjugation moieties, and tautomerism is revealed, explaining why a similar effect is not obtained for MBA. This approach results in a simpler method aimed to improve the photocatalytic effect of MIL-125-NH_2_ compared to preparations of more complex systems involving assembly with other semiconductors. The combination of these MOF nanoparticles and cellulose nanofibers leads to the formation of bionanocomposites, conforming the new materials as self-supporting, flexible, and transparent films. The incorporation of the MOF nanoparticles with loadings below 30% enhances the mechanical properties of CNF, showing an increase in the Young’s modulus from 1.3 GPa in CNF to 7.5 GPa in the film with 20% MOF loading. As a proof of concept, we have confirmed the antibacterial activity of the resultant films against S. aureus, showing their functionality as biocides by photocatalysis under visible light. The evaluated bionanocomposites incorporating the pristine or the DBA-grafted MOF present a percentage of reduction of the bacterial growth over 50% under visible light. Nevertheless, the remaining porosity of the grafted MOF could be loaded with organic biocide molecules, thus potentially enhancing their antibacterial activity. Although a systematic study in terms of the molecule grafting, radiation intensity, or wavelength range would be necessary to improve the inhibition values, the results of this preliminary assay demonstrate the applicability of the developed MOF/CNF materials in photocatalytic antimicrobial therapy.

## Supplementary Material



## Notes

Methods of preparation of cellulose nanofibers (CNF) from wood pulp and their characterization; scheme of preparation of MOF/CNF films; diagram of the film probes for mechanical testing; film sizes for mechanical testing; XRD diffractograms of MIL-125-NH_2_; FESEM images of MIL-125-NH_2_; N_2_ adsorption isotherms at 77 K of MIL-125-NH_2_; BET areas of the MOF samples; FTIR spectra of the grafted MOF samples; N_2_ adsorption isotherms and BET areas of the grafted MOF samples; bandgap energies of the different modified MOFs; XRD diffractograms of the grafted MOF samples; charge difference (Δ*Q*) between each atomic species in its bulk phase and the isolated atoms for the grafted molecule; projected density of states on N and C atoms of grafted MOF; XRD diffractograms and FTIR spectra of MIL-125-NH_2_/CNF film with different MOF loadings; UV–vis absorbance spectra of MIL-125-NH_2_/CNF films; FESEM cross-section images of MOF_20%/CNF films; mechanical properties of MIL-125-NH_2_/CNF films; agar plates showing the antibacterial effect against Staphylococcus aureus for selected samples under dark and light conditions; XRD patterns of bionanocomposite films before and after the performance of the antibacterial tests (PDF)

## References

[1] Davies J., Davies D. (2010). Origins and Evolution of Antibiotic Resistance. Microbiol Mol. Bio Rev..

[2] Levy S. B., Marshall B. (2004). Antibacterial Resistance
Worldwide: Causes, Challenges
and Responses. Nat. Med..

[3] Hutchings M. I., Truman A. W., Wilkinson B. (2019). Antibiotics: Past, Present and Future. Curr. Opin. Microbiol..

[4] Fick J., Söderström H., Lindberg R. H., Phan C., Tysklind M., Larsson D. G. J. (2009). Contamination
of Surface, Ground,
and Drinking Water from Pharmaceutical Production. Environ. Toxicol. Chem..

[5] El-Shahat M., El-Sofany W. I., Soliman A.-G. A., Hasanin M. (2022). Imidazolotriazine,
and Imidazole-Pyrazole Hybrid Derivatives as Promising Antimicrobial
Agents. J. Mol. Struct..

[6] El-Sofany W. I., Flefel E. M., Darwesh O. M., El-Shahat M. (2022). Newly Synthesized
Imidazolotriazole Boosting the Antimicrobial Performance Based on
New Fused Spirothiazolidine Framework Analogs. J. Iran. Chem. Soc..

[7] El-Shahat M., Tawfek N., El-Sofany W. I. (2025). Design,
Synthesis, Antibacterial,
and Antifungal Evaluation of a New Series of Quinazoline –
Thiazole and/or Quinazoline – Triazole Hybrids as Bioactive
Heterocycles. Chem. Biodiversity.

[8] Bedair H. M., Hamed M., Mansour F. R. (2024). New Emerging Materials
with Potential
Antibacterial Activities. App. Microbiol. Biotechnol..

[9] Zhang X., Peng F., Wang D. (2022). MOFs and MOF-Derived
Materials for
Antibacterial Application. J. Funct. Biomater..

[10] He J., Hong M., Xie W., Chen Z., Chen D., Xie S. (2022). Progress and Prospects
of Nanomaterials against Resistant Bacteria. J. Controlled Release.

[11] Faghani G., Azarniya A. (2024). Emerging Nanomaterials for Novel
Wound Dressings: From
Metallic Nanoparticles and MXene Nanosheets to Metal-Organic Frameworks. Heliyon.

[12] Bag N., Bardhan S., Roy S., Roy J., Mondal D., Guo B., Das S. (2023). Nanoparticle-Mediated Stimulus-Responsive Antibacterial
Therapy. Biomater. Sci..

[13] Ran B., Ran L., Wang Z., Liao J., Li D., Chen K., Cai W., Hou J., Peng X. (2023). Photocatalytic Antimicrobials: Principles,
Design Strategies, and Applications. Chem. Rev..

[14] Jiang S., Ma B. C., Huang W., Kaltbeitzel A., Kizisavas G., Crespy D., Zhang K. A. I., Landfester K. (2018). Visible Light
Active Nanofibrous Membrane for Antibacterial Wound Dressing. Nanoscale Horiz..

[15] Rai M., Yadav A., Gade A. (2009). Silver Nanoparticles
as a New Generation
of Antimicrobials. Biotechnol. Adv..

[16] Pérez-Carvajal J., Lalueza P., Casado C., Téllez C., Coronas J. (2012). Layered Titanosilicates
JDF-L1 and AM-4 for Biocide
Applications. Appl. Clay Sci..

[17] Zhou H.-C., Long J. R., Yaghi O. M. (2012). Introduction
to Metal–Organic
Frameworks. Chem. Rev..

[18] Farha O. K., Eryazici I., Jeong N. C., Hauser B. G., Wilmer C. E., Sarjeant A. A., Snurr R. Q., Nguyen S. T., Yazaydin A. Ö., Hupp J. T. (2012). Metal-Organic Framework
Materials with Ultrahigh Surface
Areas: Is the Sky the Limit?. J. Am. Chem. Soc..

[19] Freund R., Canossa S., Cohen S. M., Yan W., Deng H., Guillerm V., Eddaoudi M., Madden D. G., Fairen-Jimenez D., Lyu H. (2021). 25 Years of Reticular
Chemistry. Angew. Chem. Int. Ed..

[20] Guo L., Kong W., Che Y., Liu C., Zhang S., Liu H., Tang Y., Yang X., Zhang J., Xu C. (2024). Research Progress
on Antibacterial Applications of Metal-Organic Frameworks and Their
Biomacromolecule Composites. Int. J. Biol. Macromol..

[21] Wang Q., Gao Q., Al-Enizi A. M., Nafady A., Ma S. (2020). Recent Advances in
MOF-Based Photocatalysis: Environmental Remediation under Visible
Light. Inorg. Chem. Front..

[22] Navalón S., Dhakshinamoorthy A., Álvaro M., Ferrer B., García H. (2023). Metal–Organic
Frameworks as Photocatalysts for Solar-Driven Overall Water Splitting. Chem. Rev..

[23] Li R., Chen T., Pan X. (2021). Metal-Organic-Framework-Based Materials
for Antimicrobial Applications. ACS Nano.

[24] Harding J. L., Reynolds M. M. (2012). Metal Organic Frameworks
as Nitric Oxide Catalysts. J. Am. Chem. Soc..

[25] Darder M., Karan A., del Real G., DeCoster M. A. (2020). Cellulose-Based
Biomaterials Integrated with Copper-Cystine Hybrid Structures as Catalysts
for Nitric Oxide Generation. Mater. Sci. Eng.:
C.

[26] Rengaraj A., Puthiaraj P., Heo N. S., Lee H., Hwang S. K., Kwon S., Ahn W. S., Huh Y. S. (2017). Porous NH2-MIL-125
as an Efficient Nano-Platform for Drug Delivery, Imaging, and ROS
Therapy Utilized Low-Intensity Visible Light Exposure System. Colloids Surf., B.

[27] Emam H. E., El-Shahat M., Abdelhameed R. M. (2021). Observable Removal of Pharmaceutical
Residues by Highly Porous Photoactive Cellulose Acetate@MIL-MOF Film. J. Hazard. Mater..

[28] Doan T. D., Vu N.-N., Hoang T. L. G., Nguyen-Tri P. (2025). Metal-Organic
Framework (MOF)-Based Materials for Photocatalytic Antibacterial Applications. Coord. Chem. Rev..

[29] Sun X., Pan W., Wang G., Liu S., Zhang Y., Huang J., Zhang H., Wang J., Xi S., Luo T. (2023). Ag Nanoparticle
and Ti–MOF Cooperativity for Efficient Inactivation of E. Coli in Water. ACS Appl.
Mater. Interfaces.

[30] Rehan M., Montaser A. S., El-Shahat M., Abdelhameed R. M. (2024). Decoration
of Viscose Fibers with Silver Nanoparticle-Based Titanium-Organic
Framework for Use in Environmental Applications. Environ. Sci. Pollut. Res. Int..

[31] Abdelhameed R. M., El-Shahat M. (2023). Removable
Visible-Light Photocatalysts for Nitro-Group
Reduction Constructed from Palladium Decorated Ti-MOFs Integrate onto
Fibers. Mater. Sci. Eng. B.

[32] Abdelhameed R. M., El-Shahat M., Emam H. E. (2020). Employable Metal
(Ag & Pd)@MIL-125-NH2@cellulose
Acetate Film for Visible-Light Driven Photocatalysis for Reduction
of Nitro-Aromatics. Carbohydr. Polym..

[33] Abdelhameed R. M., Abu-Elghait M., El-Shahat M. (2022). Engineering Titanium-Organic Framework
Decorated Silver Molybdate and Silver Vanadate as Antimicrobial, Anticancer
Agents, and Photo-Induced Hydroxylation Reactions. J. Photochem. Photobiol. A Chem..

[34] Abdelhameed R. M., Al Kiey S. A., Wassel A. R., El-Shahat M. (2021). Silver Chromate
Doped Ti-Based Metal Organic Framework: Synthesis, Characterization,
and Electrochemical and Selective Photocatalytic Reduction Properties. New J. Chem..

[35] Abdelhameed R. M., Darwesh O. M., El-Shahat M. (2023). Titanium-Based
Metal-Organic Framework
Capsulated with Magnetic Nanoparticles: Antimicrobial and Photocatalytic
Degradation of Pesticides. Microporous Mesoporous
Mater..

[36] Wani M. Y., Bashir N., Ahmad S., Rehman M., Shah S. A., Rehman Beig S. U. (2025). Integrating
Ni-MOF/g-C3N4/Chitosan Derived S-Scheme
Photocatalyst for Efficient Visible Light Photodegradation of Tetracycline
and Antibacterial Activities. Environ. Res..

[37] Abdelhameed R. M., El-Shahat M., Abd El-Ghaffar M. A. (2022). Boosting the Photocatalytic Activity
of Ti-MOF via Emerging with Metal Phthalocyanine to Degrade Hazard
Textile Pigments. J. Alloys Compd..

[38] Duan J., Song Y., Sun M., Zhang J., Zhang H., Duan J. (2025). A Micro Flower-Rod-Shaped
NH2-MIL/ZnIn2S4 with Efficient Photocatalytic
Antibacterial Performance via in-Situ Composite on Indium-Based MIL
Organic Framework. J. Photochem. Photobiol.
A Chem..

[39] Abdelhameed R. M., Abu-Elghait M., El-Shahat M. (2020). Hybrid Three MOFs Composites (ZIF-67@ZIF-8@MIL-125-NH2):
Enhancement the Biological and Visible-light Photocatalytic Activity. J. Environ. Chem. Eng..

[40] Abdelhameed R. M., El-Shahat M. (2019). Fabrication of ZIF-67@MIL-125-NH2
Nanocomposite with
Enhanced Visible Light Photoreduction Activity. J. Environ. Chem. Eng..

[41] Dan-Hardi M., Serre C., Frot T., Rozes L., Maurin G., Sanchez C., Férey G. (2009). A New Photoactive
Crystalline Highly
Porous Titanium­(IV) Dicarboxylate. J. Am. Chem.
Soc..

[42] Hendon C. H., Tiana D., Fontecave M., Sanchez C., D’Arras L., Sassoye C., Rozes L., Mellot-Draznieks C., Walsh A. (2013). Engineering the Optical Response of the Titanium-MIL-125 Metal-Organic
Framework through Ligand Functionalization. J. Am. Chem. Soc..

[43] Cavka J. H., Jakobsen S., Olsbye U., Guillou N., Lamberti C., Bordiga S., Lillerud K. P. (2008). A New Zirconium
Inorganic Building
Brick Forming Metal Organic Frameworks with Exceptional Stability. J. Am. Chem. Soc..

[44] Wu Z., Huang X., Zheng H., Wang P., Hai G., Dong W., Wang G. (2018). Aromatic Heterocycle-Grafted
NH2-MIL-125­(Ti)
via Conjugated Linker with Enhanced Photocatalytic Activity for Selective
Oxidation of Alcohols under Visible Light. Appl.
Catal., B.

[45] Muelas-Ramos V., Peñas-Garzón M., Rodriguez J. J., Bedia J., Belver C. (2022). Solar Photocatalytic Degradation
of Emerging Contaminants Using NH2-MIL-125 Grafted by Heterocycles. Sep. Purif. Technol..

[46] Marshall R. J., Forgan R. S. (2016). Postsynthetic Modification of Zirconium Metal-Organic
Frameworks. Eur. J. Inorg. Chem..

[47] Luan Y., Qi Y., Gao H., Andriamitantsoa R. S., Zheng N., Wang G. (2015). A General
Post-Synthetic Modification Approach of Amino-Tagged Metal–Organic
Frameworks to Access Efficient Catalysts for the Knoevenagel Condensation
Reaction. J. Mater. Chem. A.

[48] Kandiah M., Usseglio S., Svelle S., Olsbye U., Lillerud K. P., Tilset M. (2010). Post-Synthetic Modification
of the Metal–Organic
Framework Compound UiO-66. J. Mater. Chem..

[49] Peikert K., Hoffmann F., Fröba M. (2012). Amino Substituted
Cu3­(Btc)­2: A New
Metal–Organic Framework with a Versatile Functionality. Chem. Commun..

[50] Hintz H., Wuttke S. (2014). Postsynthetic Modification of an Amino-Tagged MOF Using
Peptide Coupling Reagents: A Comparative Study. Chem. Commun..

[51] Wang Z., Tanabe K. K., Cohen S. M. (2010). Tuning
Hydrogen Sorption Properties
of Metal–Organic Frameworks by Postsynthetic Covalent Modification. Chemi Eur. J..

[52] Garibay S. J., Cohen S. M. (2010). Isoreticular Synthesis
and Modification of Frameworks
with the UiO-66 Topology. Chem. Commun..

[53] Soilis Z. M., Choi T. H., Brennan J., Frontiera R. R., Johnson J. K., Rosi N. L. (2023). Ligand Chromophore
Modification Approach
for Predictive Incremental Tuning of Metal–Organic Framework
Color. Chem. Mater..

[54] Luo H. B., Lin F. R., Liu Z. Y., Kong Y. R., Idrees K. B., Liu Y., Zou Y., Farha O. K., Ren X. M. (2023). MOF-Polymer Mixed
Matrix Membranes as Chemical Protective Layers for Solid-Phase Detoxification
of Toxic Organophosphates. ACS Appl. Mater.
Interfaces.

[55] Hsieh C. T., Ariga K., Shrestha L. K., Hsu S. H. (2021). Development of MOF
Reinforcement for Structural Stability and Toughness Enhancement of
Biodegradable Bioinks. Biomacromolecules.

[56] Abdelhamid H. N., Mathew A. P. (2022). Cellulose–Metal Organic Frameworks (CelloMOFs)
Hybrid Materials and Their Multifaceted Applications: A Review. Coord. Chem. Rev..

[57] Tignol P., Pimenta V., Dupont A.–L., Carvalho S., Mohtar A. A., Severino M. I., Nouar F., Pinto M. L., Serre C., Lavédrine B. (2024). A Versatile Shaping Method of Very-High Loading Porous
Solids Paper Adsorbent Composites. Small Methods.

[58] Zhou S., Gao J., Zhu J., Peng D., Zhang Y., Zhang Y. (2020). Self-Cleaning,
Antibacterial Mixed Matrix Membranes Enabled by Photocatalyst Ti-MOFs
for Efficient Dye Removal. J. Membr. Sci..

[59] Qian L., Lei D., Duan X., Zhang S., Song W., Hou C., Tang R. (2018). Design and
Preparation of Metal-Organic Framework Papers with Enhanced
Mechanical Properties and Good Antibacterial Capacity. Carbohydr. Polym..

[60] Duan C., Meng J., Wang X., Meng X., Sun X., Xu Y., Zhao W., Ni Y. (2018). Synthesis of Novel
Cellulose- Based
Antibacterial Composites of Ag Nanoparticles@ Metal-Organic Frameworks@
Carboxymethylated Fibers. Carbohydr. Polym.

[61] Li T., Chen C., Brozena A. H., Zhu J. Y., Xu L., Driemeier C., Dai J., Rojas O. J., Isogai A., Wågberg L., Hu L. (2021). Developing Fibrillated Cellulose
as a Sustainable Technological Material. Nature.

[62] Nechyporchuk O., Belgacem M. N., Bras J. (2016). Production
of Cellulose Nanofibrils:
A Review of Recent Advances. Industrial Crops
and Products.

[63] Saito T., Kimura S., Nishiyama Y., Isogai A. (2007). Cellulose Nanofibers
Prepared by TEMPO-Mediated Oxidation of Native Cellulose. Biomacromolecules.

[64] Isogai A., Saito T., Fukuzumi H. (2011). TEMPO-Oxidized
Cellulose Nanofibers. Nanoscale.

[65] Thakur V., Guleria A., Kumar S., Sharma S., Singh K. (2021). Recent Advances
in Nanocellulose Processing, Functionalization and Applications: A
Review. Mater. Adv..

[66] Trache D., Tarchoun A. F., Derradji M., Hamidon T. S., Masruchin N., Brosse N., Hussin M. H. (2020). Nanocellulose:
From Fundamentals
to Advanced Applications. Front. Chem..

[67] Norrrahim M. N. F., Nurazzi N. M., Jenol M. A., Farid M. A. A., Janudin N., Ujang F. A., Yasim-Anuar T. A. T., Syed Najmuddin S. U. F., Ilyas R. A. (2021). Emerging Development
of Nanocellulose as an Antimicrobial
Material: An Overview. Mater. Adv..

[68] Martin C., Borreguero C., Kennes K., Van der Auweraer M., Hofkens J., de Miguel G., García-Frutos E. M. (2017). Simple
Donor–Acceptor Luminogen Based on an Azaindole Derivative as
Solid-State Emitter for Organic Light-Emitting Devices. ACS Energy Lett..

[69] Makuła P., Pacia M., Macyk W. (2018). How To Correctly
Determine the Band
Gap Energy of Modified Semiconductor Photocatalysts Based on UV-Vis
Spectra. J. Phys. Chem. Lett..

[70] Soler J. M., Artacho E., Gale J. D., García A., Junquera J., Ordejón P., Sánchez-Portal D. (2002). The SIESTA
method for ab initio order- N materials simulation. J. Phys.: Condens. Matter.

[71] García A., Papior N., Akhtar A., Artacho E., Blum V., Bosoni E., Brandimarte P., Brandbyge M., Cerdá J. I., Corsetti F. (2020). Siesta:
Recent Developments
and Applications. J. Chem. Phys..

[72] Kleinman L., Bylander D. M. (1982). Efficacious Form
for Model Pseudopotentials. Phys. Rev. Lett..

[73] Troullier N., Martins J. L. (1991). Efficient Pseudopotentials for Plane-Wave Calculations. Phys. Rev. B.

[74] Perdew J. P., Burke K., Ernzerhof M. (1996). Generalized
Gradient Approximation
Made Simple. Phys. Rev. Lett..

[75] Grimme S., Antony J., Ehrlich S., Krieg H. (2010). A Consistent and Accurate
Ab Initio Parametrization of Density Functional Dispersion Correction
(DFT-D) for the 94 Elements H-Pu. J. Chem. Phys..

[76] Mulliken R. S. (1955). Electronic
Population Analysis on LCAO–MO Molecular Wave Functions. I. J. Chem. Phys..

[77] Chambers M. B., Wang X., Ellezam L., Ersen O., Fontecave M., Sanchez C., Rozes L., Mellot-Draznieks C. (2017). Maximizing
the Photocatalytic Activity of Metal-Organic Frameworks with Aminated-Functionalized
Linkers: Substoichiometric Effects in MIL-125-NH2. J. Am. Chem. Soc..

[78] Peñas-Garzón M., Gómez-Avilés A., Álvarez-Conde J., Bedia J., García-Frutos E. M., Belver C. (2023). Azaindole
Grafted Titanium Dioxide for the Photodegradation of Pharmaceuticals
under Solar Irradiation. J. Colloid Interface
Sci..

[79] Socrates, G. Infrared and Raman Characteristic Group Frequencies: tables and Charts, 3rd ed.; Wiley, 2004.

[80] Arunkumar T., Castelino E., Lakshmi T., Mulky L., Selvanathan S. P., Tahir M. (2024). Metal–Organic Frameworks in
Antibacterial Disinfection: A
Review. ChemBioEng Rev..

[81] Suna P., Misra P. K., Panigrahi S. (2019). Substituted
Benzylideneanilines:
A Family of Solvatochromic Probes. Spectrochim.
Acta, Part A.

[82] Kandambeth S., Mallick A., Lukose B., Mane M. V., Heine T., Banerjee R. (2012). Construction of Crystalline 2D Covalent Organic Frameworks
with Remarkable Chemical (Acid/Base) Stability via a Combined Reversible
and Irreversible Route. J. Am. Chem. Soc..

[83] Demir E. C., McDermott M. T., Kim C. L., Ayranci C. (2023). Towards Better Understanding
the Stiffness of Nanocomposites via Parametric Study of an Analytical
Model Modeling Parameters and Experiments. J.
Compos. Mater..

[84] Liang J., Zou G., Gu C., Tao S., Guo L., Tang C., Zhang J., Deng Z., Chen Y. (2023). Study on Skin Infection
Model of Staphylococcus Aureus Based on Analytic Hierarchy Process
and Delphi Method. Heliyon.

[85] Khan Z. A., Goda E. S., Ur Rehman A., Sohail M. (2021). Selective Antimicrobial
and Antibiofilm Activity of Metal–Organic Framework NH2-MIL-125
against Staphylococcus Aureus. Mater. Sci. Eng.
B.

[86] Zheng S., Bawazir M., Dhall A., Kim H.–E., He L., Heo J., Hwang G. (2021). Implication
of Surface Properties,
Bacterial Motility, and Hydrodynamic Conditions on Bacterial Surface
Sensing and Their Initial Adhesion. Front. Bioeng.
Biotechnol..

[87] Yin N., Santos T. M. A., Auer G. K., Crooks J. A., Oliver P. M., Weibel D. B. (2014). Bacterial Cellulose
as a Substrate for Microbial Cell
Culture. Appl. Environ. Microbiol..

[88] Dou J., Ilina P., Cruz C. D., Nurmi D., Vidarte P. Z., Rissanen M., Tammela P., Vuorinen T. (2023). Willow Bark-Derived
Material with Antibacterial and Antibiofilm Properties for Potential
Wound Dressing Applications. J. Agric. Food
Chem..

[89] Luo Y., Li B., Liu X., Zheng Y., Wang E., Li Z., Cui Z., Liang Y., Zhu S., Wu S. (2022). Simultaneously
Enhancing
the Photocatalytic and Photothermal Effect of NH2-MIL-125-GO-Pt Ternary
Heterojunction for Rapid Therapy of Bacteria-Infected Wounds. Bioact. Mater..

[90] Arenas-Vivo A., Amariei G., Aguado S., Rosal R., Horcajada P. (2019). An Ag-Loaded
Photoactive Nano-Metal Organic Framework as a Promising Biofilm Treatment. Acta Biomater..

