# Supplementary material for: Modulated Optoelectronic Properties of MOF/CNF Bionanocomposite Films for Bacterial Growth Control under Visible Light
Source: ACS Appl Mater Interfaces. 2025 May 10;17(20):30006–18. doi: 10.1021/acsami.5c04982 (PMC12100602; doi:10.1021/acsami.5c04982)
Supplement: Supplementary file 1 [file am5c04982_si_001.pdf]

Supporting Information

Modulated optoelectronic properties of MOF/CNF bionanocomposite films for bacterial growth control under visible light

Joab D. Guerrero[a], Raquel Martín-Sampedro[b], Ramón Cuadrado[a], Iván Llano[a], Eva M. García-Frutos[a], Pilar Aranda[a], David Ibarra[b], María E. Eugenio[b], Luis Vázquez[a], Javier Pérez-Carvajal*[,a], and Margarita Darder*[,a]

[a] Instituto de Ciencia de Materiales de Madrid (ICMM, CSIC). C/ Sor Juana Inés de la Cruz, 3, 28049 Madrid, Spain. *E-mail: jperez@icmm.csic.es, darder@icmm.csic.es

[b] Instituto de Ciencias Forestales (ICIFOR), INIA-CSIC, Ctra. de La Coruña, km 7,5, 28040 Madrid, Spain

Corresponding authors: jperez@icmm.csic.es, darder@icmm.csic.es

Preparation of cellulose nanofibers (CNF)

Cellulose nanofibers (CNF) were obtained by TEMPO-mediated oxidation of cellulose pulp followed by microfluidization. The process was carried out under alkaline pH conditions using a methodology previously described by Saito et al.[1] Cellulose pulp (10 g) was dispersed in distilled water containing TEMPO (0.016 g/g of cellulose) and NaBr (0.1 g/g of cellulose). The mixture was stirred for 15 minutes to ensure proper dispersion of the reagents. Then, a 15% sodium hypochlorite (NaClO) solution was added dropwise to the slurry. The volume of NaClO added was calculated to be 5 mmol/g of cellulose. The pH was maintained at 10 by adding drops of a 0.5 M NaOH solution. The oxidation process was considered complete when the pH remained constant at 10. The oxidized fibers were filtered and washed with distilled water five times. A significant change in the conformation can be seen from the initial cellulose pulp dispersed in water by mechanical stirring to the cellulose dispersion obtained after the oxidation reaction.

The TEMPO-catalyzed oxidation results in the selective oxidation of the primary C6 hydroxyl groups of the cellulose chains to carboxyl groups and modifies the surface charge of the cellulose fibers. This generates an electrostatic repulsion among them, reducing the probability to conform new hydrogen bonds and improving their separation. The amount of carboxyl groups generated in the oxidation process was determined by conductometric titration with NaOH according to Besbes et al.,[2] where the relation is given by equation S1. The titration was performed in triplicate with the same starting amount of CNF and substituting the parameters from equation S1, the amount of carboxyl groups in different samples was obtained (Table S1).

$$\text{Carboxyl groups}\left(\frac{mM}{g}\right) = \frac{\text{Titrant agent (M)} \cdot \text{V}_{\text{add}}\ \text{(ml)}}{\text{CNF mass (g)}} \qquad (eq. \text{S1})$$

Table S1. Titration of CNF dispersion for determination of carboxyl groups.

Titration Sample	CNF mass (g)	Titrant agent concentration (ml)	Carboxyl groups (mmol/g)
CNF-1	$3.75 \cdot 10^{-3}$	4.63	1.235
CNF-2	$3.75 \cdot 10^{-3}$	4.64	1.237
CNF-3	$3.75 \cdot 10^{-3}$	4.63	1.235

The resulting product was subjected to a mechanical treatment using a high-pressure microfluidizer (M-110P, Microfluidics) to produce defibrillation of cellulose and reduce the diameter of the fibers. First, a previous re-homogenization treatment was carried with the Ultraturrax (T25 digital, IKA) at 18,000 rpm for 5 minutes to reduce the size of the fibers and break any agglomeration generated during the previous process. Then, the fibers were passed through the microfluidizer three times at different pressures as indicated in Table S2, until a transparent gel was collected.

Table S2. Different stages of the microfluidization process to prepare CNF.

Stage	Chamber conditions		Pressure (bar)
	**Chamber 1**	**Chamber 2**	
1	-	200 µm	1000
2	200 µm	100 µm	1000
3	200 µm	100 µm	2000

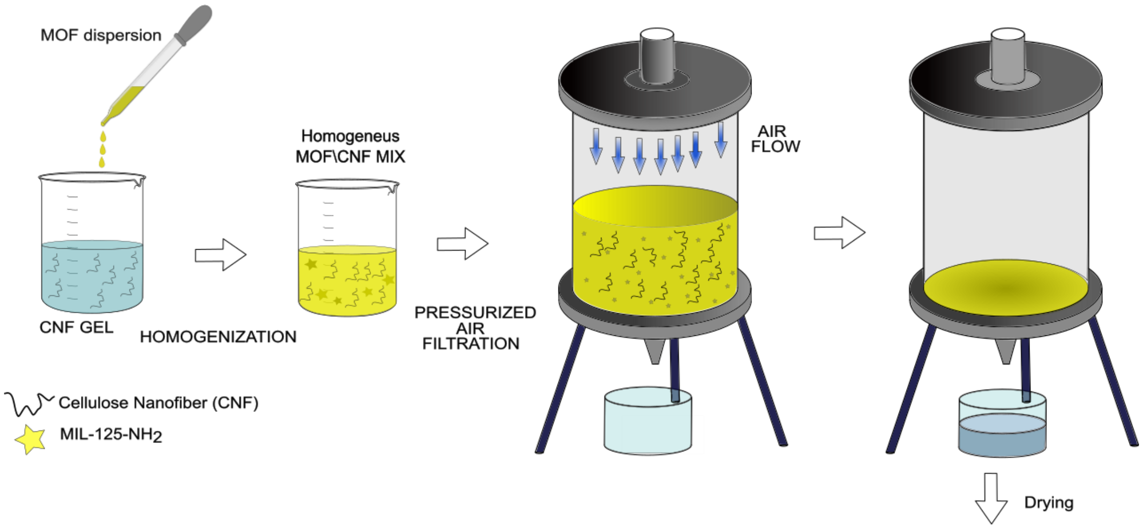

Figure S1. Schematic preparation of MOF/CNF films.

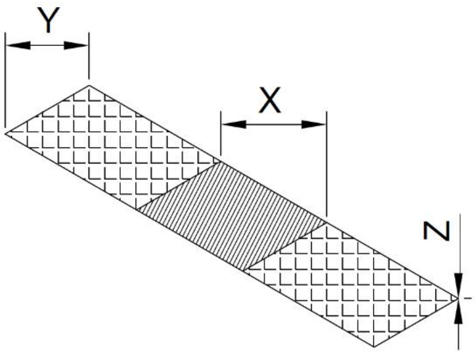

Figure S2. Film probe diagram for mechanical testing.

Table S3. Film sizes for mechanical testing.

Sample	X<br>Active length (mm)	Y<br>Width (mm)	Z<br>Thickness (μm)
CNF	12.1 ± 0.7	9.6 ± 0.7	15.5 ± 0.8
MOF_5%/CNF	12.9 ± 3.8	10.1 ± 0.5	15.3 ± 0.4
MOF_20%/CNF	22.7 ± 1.2	10.7 ± 1.0	15.6 ± 0.2
[MOF-DBA_0.5]_20%/CNF	30.8 ± 2	9.8 ± 0.7	13.7 ± 0.3

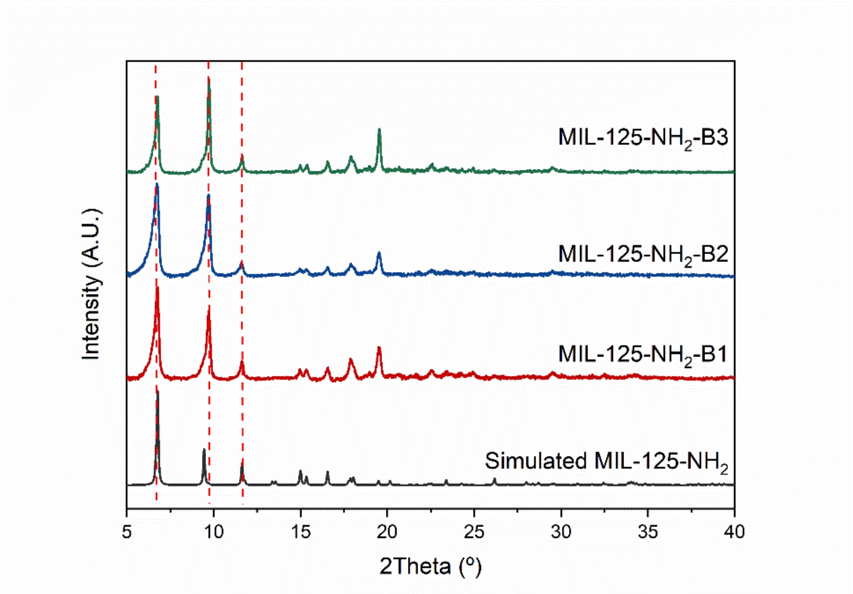

Figure S3. XRD diffractograms of different batches (B1 to B3) of MIL-125-NH2, compared with the simulated powder pattern.

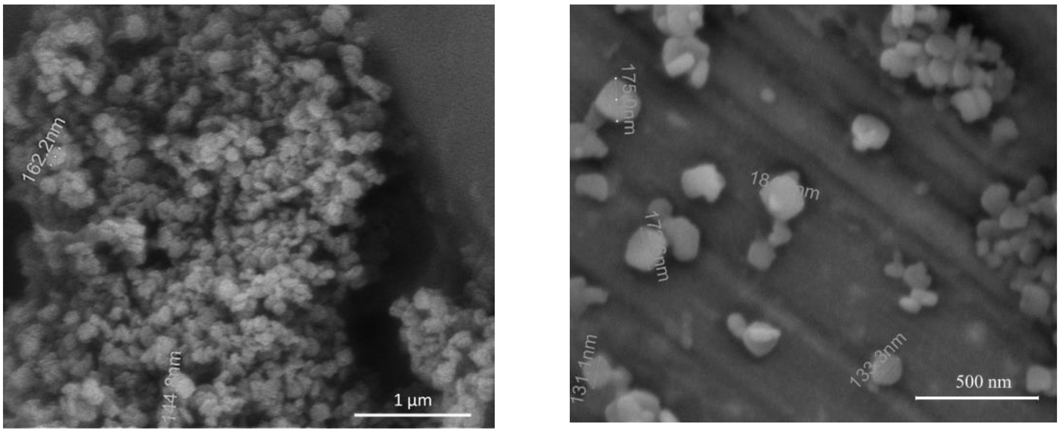

Figure S4. Representative FESEM images of MIL-125-NH2.

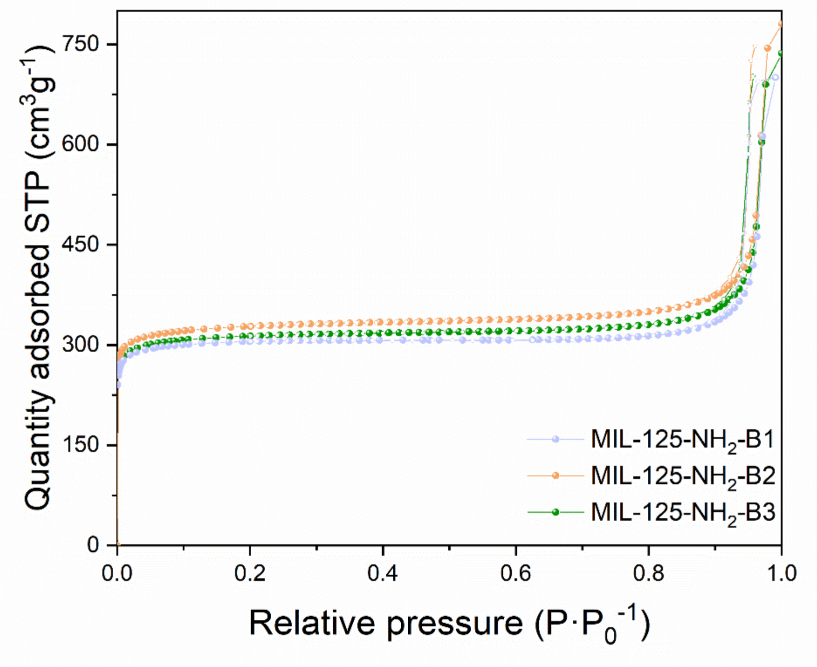

Figure S5. N_2 adsorption isotherms at 77 K of MIL-125-NH_2 from three different batches (B1 to B3).

Table S4. BET specific surface areas of MIL-125-NH_2 samples obtained from three different batches (B1 to B3).

Sample	BET area (m^2/g)	Pore volume (cm^3/g)
MIL-125-NH_2-B1	1234	0.87
MIL-125-NH_2-B2	1356	1.15
MIL-125-NH_2-B3	1348	1.01

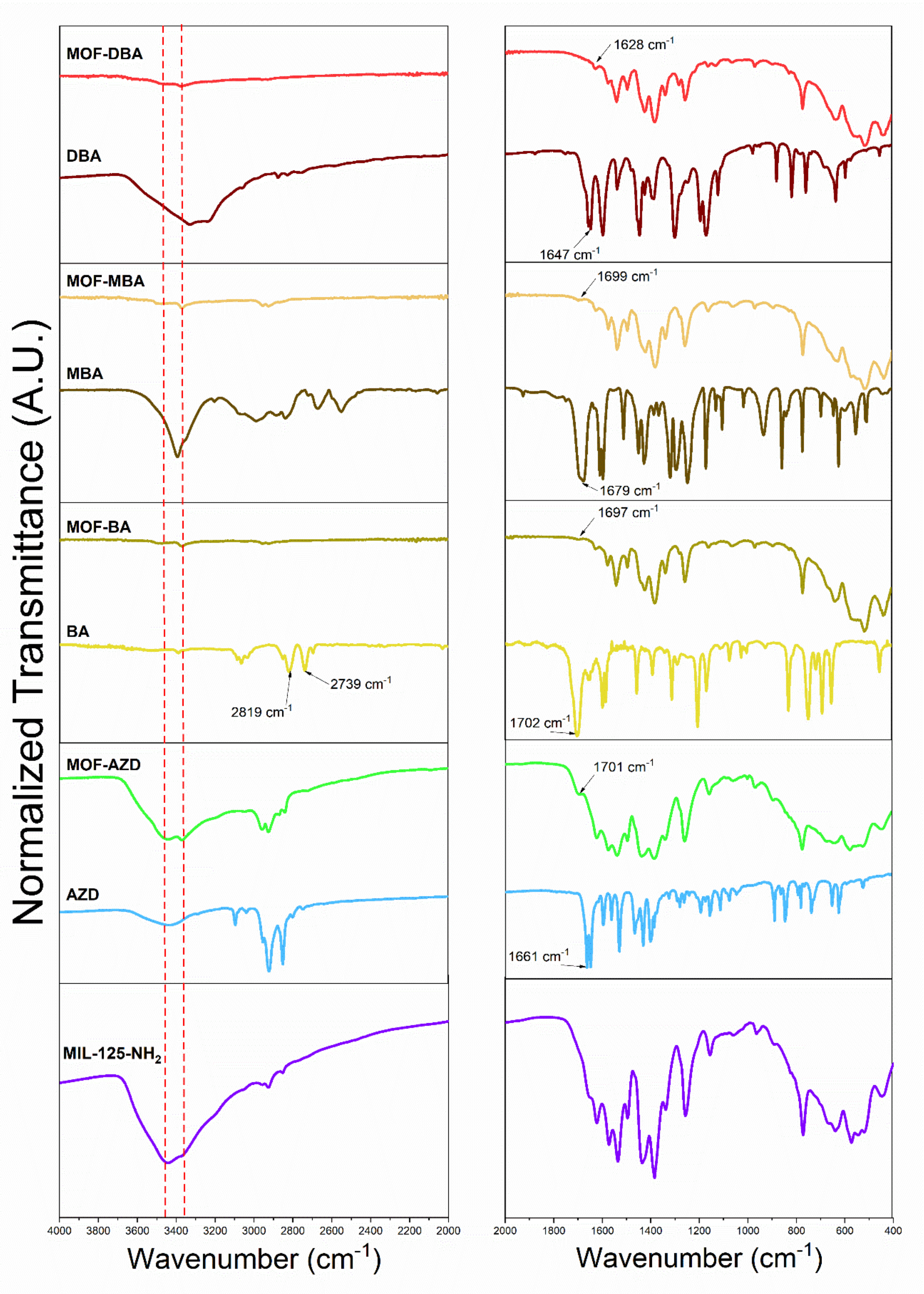

Figure S6. FTIR spectra of the samples MIL-125-NH2, AZD, MOF-AZD, BA, MOF-BA, MBA, MOF-MBA, DBA and MOF-DBA.

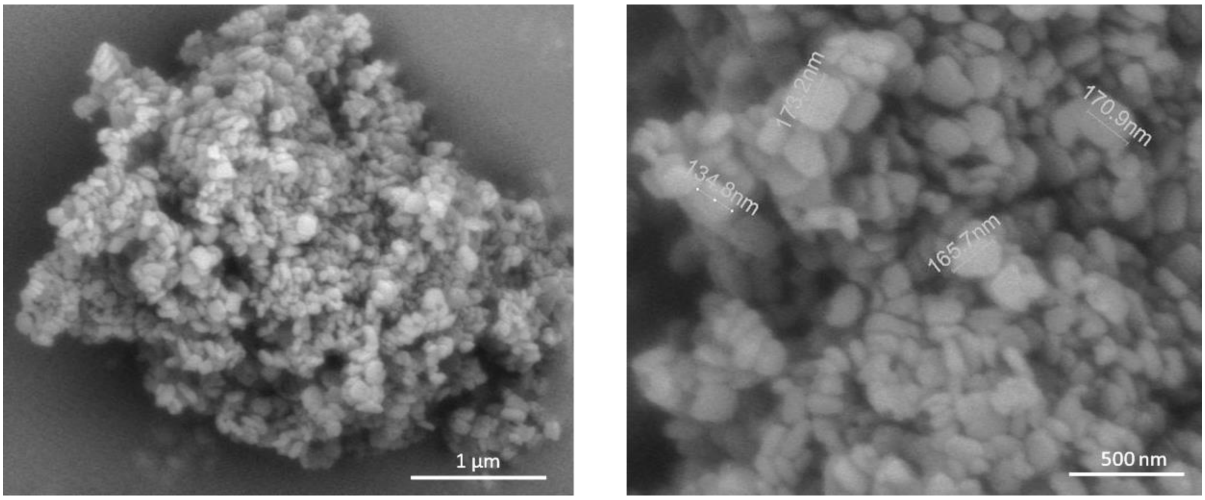

Figure S7. Representative FESEM images of MOF-DBA.

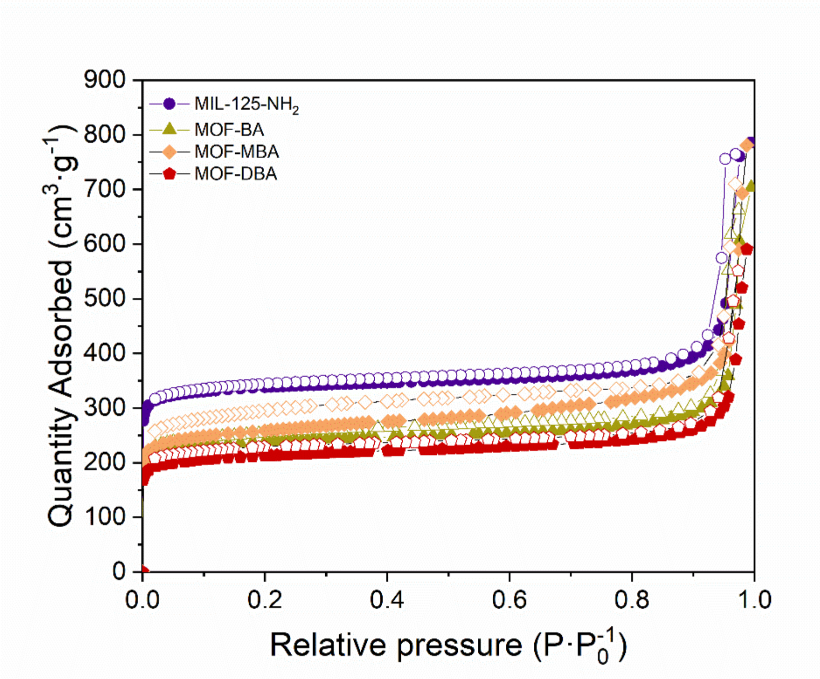

Figure S8. N_2 adsorption isotherms at 77 K of pristine MIL-125-NH_2 and MIL-125-NH_2 samples modified with the different grafting molecules: BA, MBA and DBA.

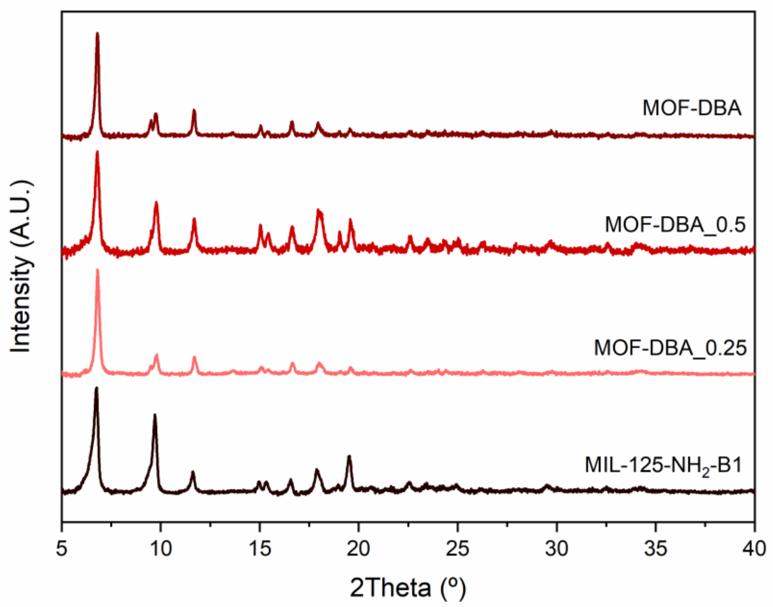

Figure S9. XRD diffractograms of MOF-DBA_0.25, MOF-DBA_0.5, MOF-DBA compared with the synthesized MIL-125-NH_2.

Table S5. BET specific surface area and pore volume of the MIL-125-NH_2 samples modified with three selected aromatic aldehydes.

Sample	BET area (m^2/g)	Pore volume (cm^3/g)
MIL-125-NH_2	1267	1.20
MOF-BA	941	1.09
MOF-MBA	997	1.21
MOF-DBA	831	0.91

Table S6. Band gap energies of the different modified MOF

Sample	Band gap energy (eV)	Reduction percentage (%)
MIL-125-NH_2	2.71	-
MOF-AZD	2.53	6.6
MOF-BA	2.48	8.2
MOF-MBA	2.5	4.4
MOF-DBA	2.10	22.5
MOF-DBA_0.5	2.08	23.2
MOF-DBA_0.25	2.14	21.0

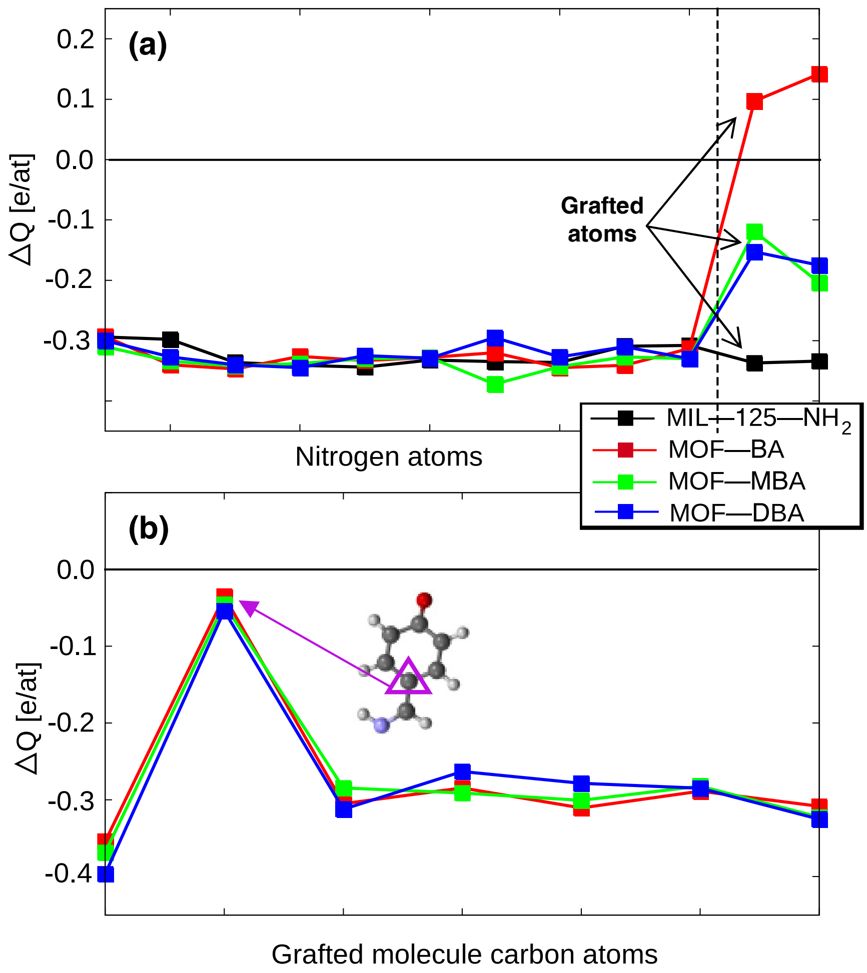

Figure S10. (Color online) Charge difference (ΔQ) between each atomic species in its bulk phase and the isolated atoms for the grafted molecules, defined as ΔQ = q_bulk − q_isol. A positive (negative) value indicates charge adsorption (reduction) relative to the isolated case. In (a), the vertical dashed black line separates the grafted N atoms from the others within the simulation unit cell. In (b), the inset provides a schematic representation of the grafted molecule pointing to the carbon atom that dramatically changes its charge.

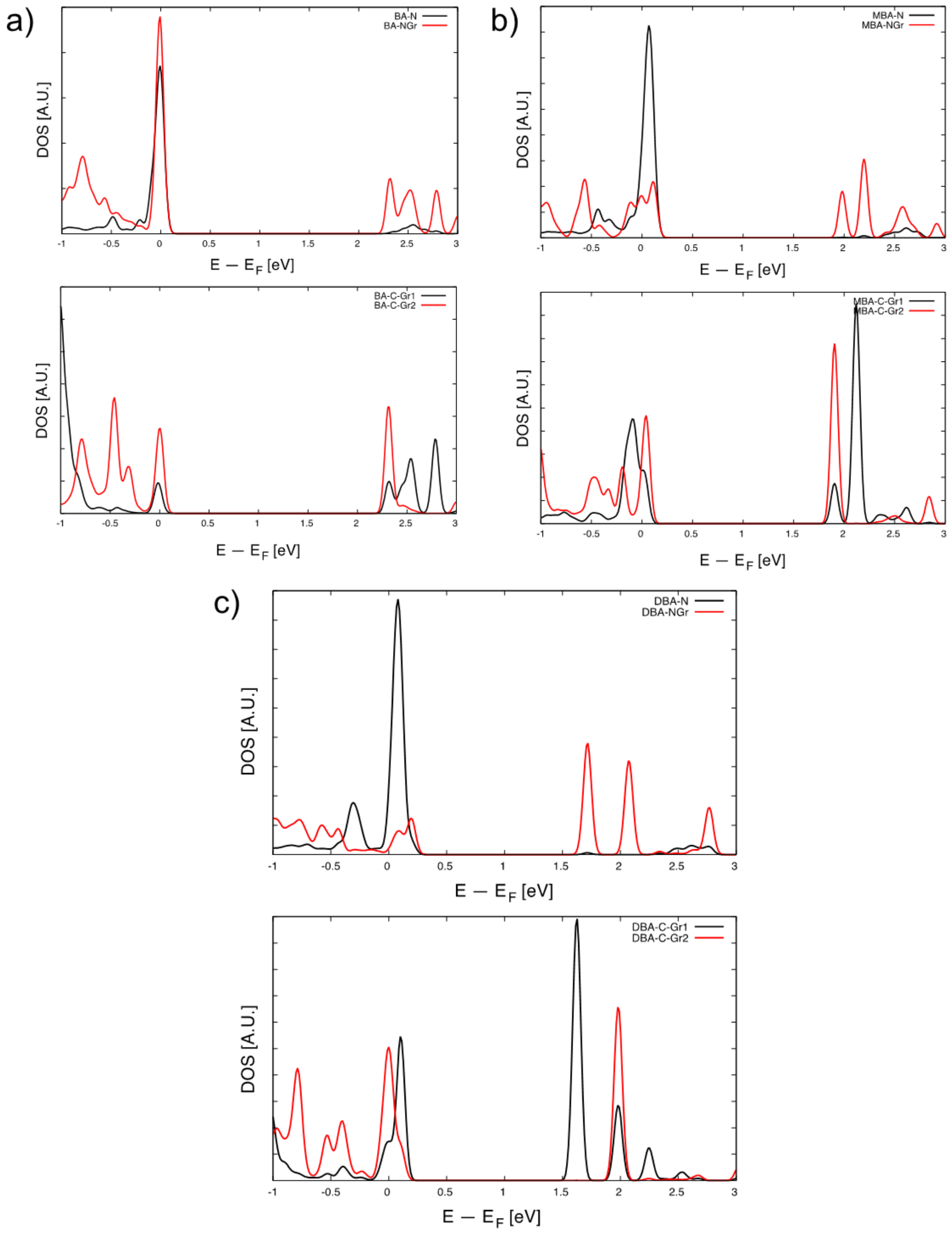

Figure S11. Density of States of N atoms (up) and C atoms (down) of MIL-125-NH_2 functionalized with BA (a), MBA (b) and DBA (c). N (Black line) refers to pristine amine nitrogen atoms where N-Gr (red line) refers to grafted N atoms on the optimized crystal structures. C-Gr refers to position of grated molecules in the crystal structures being 1 closer to titanium clusters.

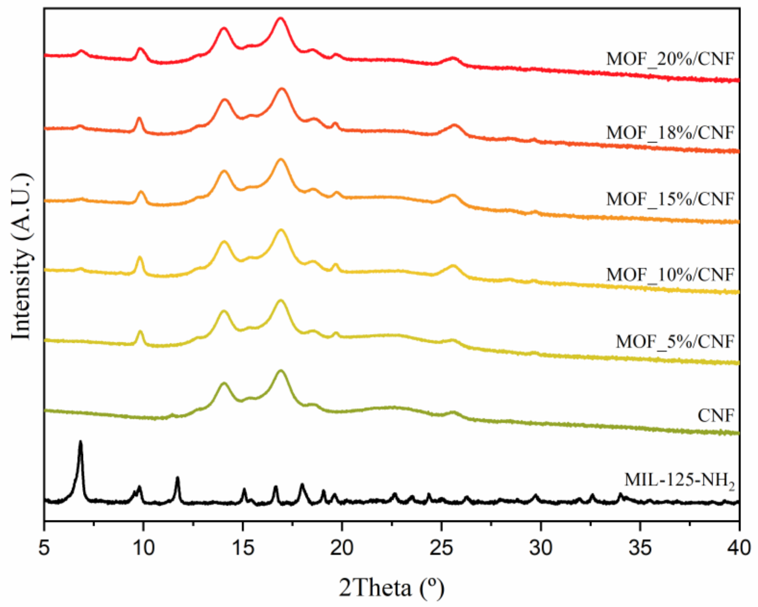

Figure S12. XRD diffractograms of MIL-125-NH_2/CNF film with different MOF loadings.

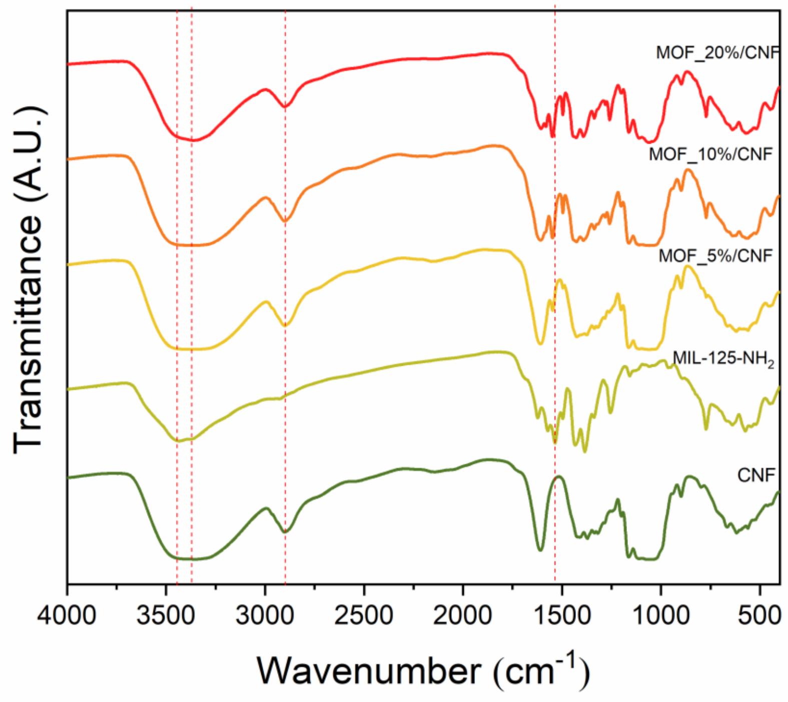

Figure S13. FTIR spectra of MIL-125-NH_2/CNF film with different MOF loadings.

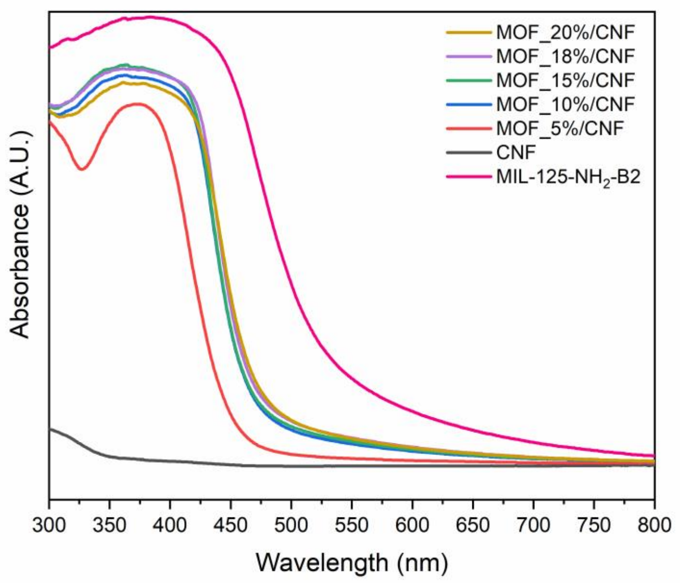

Figure S14. UV-vis absorbance spectra of MIL-125-NH_2/CNF film with different MOF loadings.

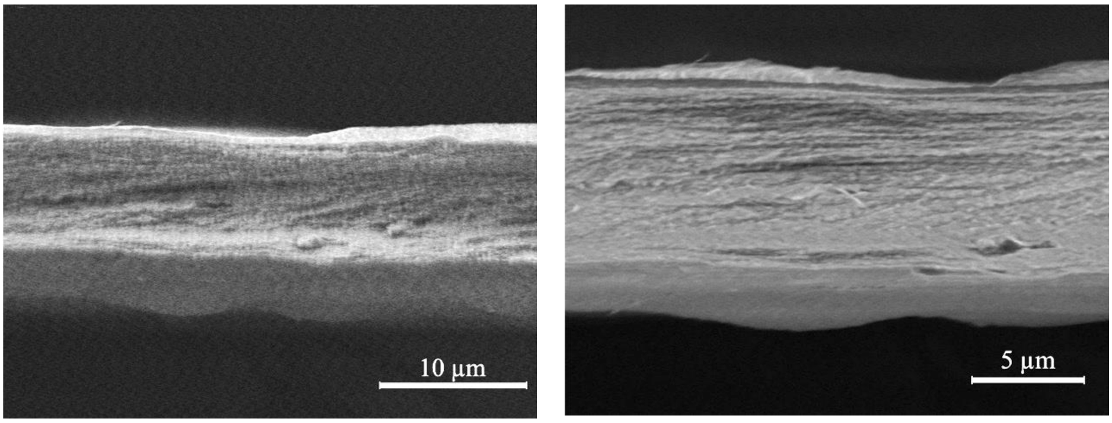

Figure S15. Representative FESEM images of cross sections of MOF_20%/CNF films at different scales.

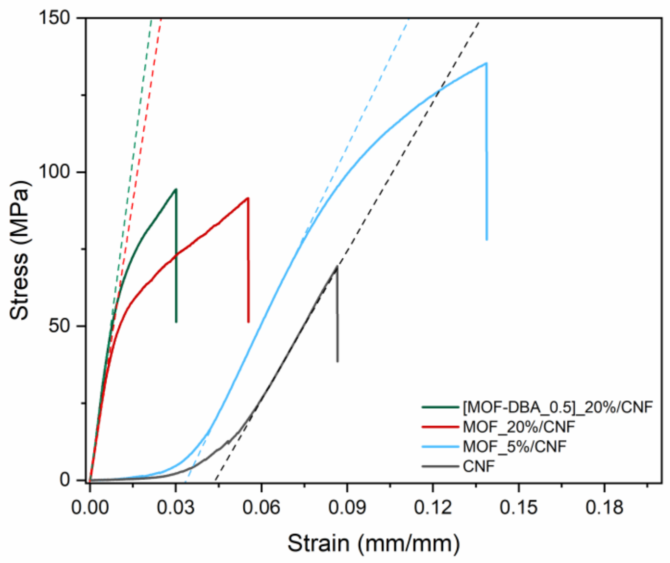

Figure S16. Mechanical properties of the MOF_5%/CNF, MOF_20%/CNF, and [MOF-DBA_0.5]_20%/CNF bionanocomposite films compared to that of pristine CNF.

a)

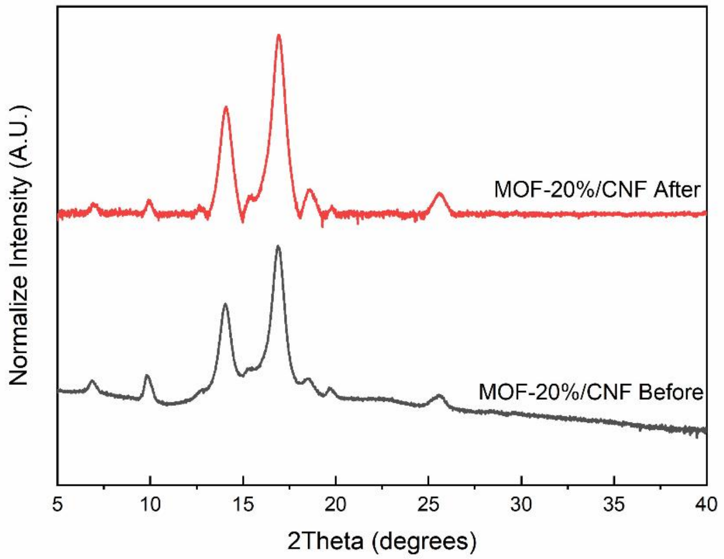

b)

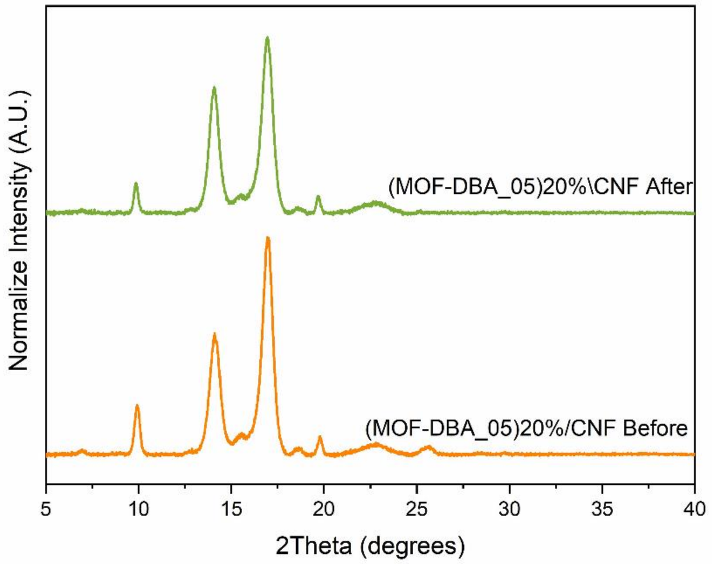

Figure S17. XRD patterns of a) MOF20%/CNF and b) (MOF-DBA_05)20%/CNF films before and after the performance of the antibacterial tests.

References

(1) Saito, T.; Kimura, S.; Nishiyama, Y.; Isogai, A. Cellulose nanofibers prepared by TEMPO-mediated oxidation of native cellulose. Biomacromolecules, 2007, 8, 2485–2491. https://doi.org/10.1021/bm0703970.

(2) Besbes, I.; Alila, S.; Boufi, S. Nanofibrillated cellulose from TEMPO-oxidized eucalyptus fibres: Effect of the carboxyl content, Carbohydrate Polymers, 2011, 84, 975-983. https://doi.org/10.1016/j.carbpol.2010.12.052.
